# Antimicrobial Susceptibility Testing of Antimicrobial Peptides to Better Predict Efficacy

**DOI:** 10.3389/fcimb.2020.00326

**Published:** 2020-07-07

**Authors:** Derry K. Mercer, Marcelo D. T. Torres, Searle S. Duay, Emma Lovie, Laura Simpson, Maren von Köckritz-Blickwede, Cesar de la Fuente-Nunez, Deborah A. O'Neil, Alfredo M. Angeles-Boza

**Affiliations:** ^1^NovaBiotics Ltd, Aberdeen, United Kingdom; ^2^Machine Biology Group, Departments of Psychiatry and Microbiology, Institute for Biomedical Informatics, Institute for Translational Medicine and Therapeutics, Perelman School of Medicine, Penn Institute for Computational Science, and Department of Bioengineering, University of Pennsylvania, Philadelphia, PA, United States; ^3^Department of Chemistry, Institute of Materials Science, University of Connecticut, Storrs, CT, United States; ^4^Institute for Biochemistry, University of Veterinary Medicine, Hanover, Germany

**Keywords:** antimicrobial peptide (AMP), antimicrobial susceptibility testing, antibiotic, antifungal, host defence peptide (HDP)

## Abstract

During the development of antimicrobial peptides (AMP) as potential therapeutics, antimicrobial susceptibility testing (AST) stands as an essential part of the process in identification and optimisation of candidate AMP. Standard methods for AST, developed almost 60 years ago for testing conventional antibiotics, are not necessarily fit for purpose when it comes to determining the susceptibility of microorganisms to AMP. Without careful consideration of the parameters comprising AST there is a risk of failing to identify novel antimicrobials at a time when antimicrobial resistance (AMR) is leading the planet toward a post-antibiotic era. More physiologically/clinically relevant AST will allow better determination of the preclinical activity of drug candidates and allow the identification of lead compounds. An important consideration is the efficacy of AMP in biological matrices replicating sites of infection, e.g., blood/plasma/serum, lung bronchiolar lavage fluid/sputum, urine, biofilms, etc., as this will likely be more predictive of clinical efficacy. Additionally, specific AST for different target microorganisms may help to better predict efficacy of AMP in specific infections. In this manuscript, we describe what we believe are the key considerations for AST of AMP and hope that this information can better guide the preclinical development of AMP toward becoming a new generation of urgently needed antimicrobials.

## Antimicrobial Resistance

Antimicrobial resistance (AMR) is a global health crisis. The over-use and inappropriate prescribing of antibiotics has set us on a path toward a post-antibiotic era where our existing armory of antibiotics will no longer be effective. Increasing numbers of microorganisms are already becoming widely resistant to existing antibiotic classes (O'Neill, 2016; Barlow, 2018; Chatterjee et al., 2018; Van Puyvelde et al., 2018; Roope et al., 2019). The need for new antimicrobial agents is more important now than it has ever been. So too is the need for better antimicrobial stewardship; prudent and appropriate use of antimicrobials.

AMR is responsible for 700,000 deaths per annum globally (Blair et al., 2015; European Commission, 2017) but this is forecast to increase to 10 million deaths annually by 2050 (killing more people than cancer and diabetes) if the measures highlighted above are not urgently implemented successfully to address drug resistant infections (O'Neill, 2016). This assessment was based on scenarios for rising drug resistance and economic growth to 2050 for six major pathogens/infectious diseases; *Klebsiella pneumoniae, Escherichia coli, Staphylococcus aureus*, malaria, tuberculosis and HIV (O'Neill, 2014). According to the Centers for Disease Control and Prevention (CDC), in the US alone over 2.8 million infections per year are caused by antibiotic-resistant bacteria, causing more than 35,000 deaths in the US per annum and US$55 billion in increased healthcare costs and lost productivity (CDC, 2019). Strikingly, in 2009–2010 in the US almost 20% of pathogens reported from Hospital-acquired infections (HAI) were multidrug-resistant (Sievert et al., 2013).

Most of the antibiotics available today are broad-spectrum molecules derived from agents that have been in use for more than 30 years and as well as “failing” through resistance, oftentimes have unintended side effects, such as toxicity toward beneficial commensal bacteria and mammalian cells, and triggering inflammatory responses (Lepore et al., 2019). Research efforts are ongoing to discover and develop new, more effective and safe antimicrobial agents that can overcome bacterial resistance mechanisms, occasionally even presenting selective activity toward single bacterial species or specific strains of bacteria (de la Fuente-Nunez et al., 2017b).

## Antimicrobial Peptides/Host Defence Peptides

Antimicrobial peptides (AMP) have potential as a new therapeutic class of antimicrobials and are one of the most promising scaffolds being explored for the generation of much-needed novel antibacterials and antifungals. The blueprint for many AMP as drugs are endogenous Host Defence Peptides (HDP); relatively small peptides (4–50 amino acid residues) that are generally positively charged and often contain an amphipathic conformation (Jiang et al., 2009; Mercer et al., 2019; Torres et al., 2019). In the context of this manuscript AMP refers to all peptides with antimicrobial properties, whereas HDP are essential innate host defence effector molecules and are amongst the “first responders” in all eukaryotic and some prokaryotic organisms to infectious challenge or an inflammation (Zasloff, 2002; Hassan et al., 2012; Mansour et al., 2014; Kang et al., 2017). As well as having direct antimicrobial activity against bacteria, fungi and parasites, HDP can modulate the host immune response, hence being termed host defence peptides (Hancock and Sahl, 2006; Mansour et al., 2014). HDP are often classified according to the structure they tend to adopt in hydrophilic/hydrophobic interfaces, such as the interface of microbial cell membranes and the extracellular environment, e. g., α-helix, β-sheet, etc. (Wang, 2015). Often, ~50% of their sequence comprises hydrophobic and aliphatic residues that facilitate interactions with and translocation across membranes to form pores or to enter cells (Jenssen et al., 2006; Fjell et al., 2011; Aoki and Ueda, 2013). HDP and the AMP derived from these scaffolds are versatile molecules with a wide diversity of structural and physicochemical properties, and are able to target microorganisms through diverse mechanisms of action, although the most common mechanism of action is membrane perturbation/lysis (Brogden, 2005; Hancock and Sahl, 2006; Le et al., 2017; Pyne et al., 2017; Kumar et al., 2018; Aisenbrey et al., 2019). For the purposes of this manuscript, we will refer to both AMP and HDP as AMP.

Achieving precise control over AMP properties and understanding how peptides behave in different environments are still challenges in the field (Naafs, 2018). The understanding of AMP features and details of their mechanism/s of action are still not clear and have been the target of many studies (Porto et al., 2018; Torres et al., 2018; Torres and de la Fuente-Nunez, 2019; Yount et al., 2019). Some of the most promising approaches to describe the role of structural and physicochemical properties on AMP antimicrobial activity are those involving computer-based strategies combined with high-throughput experiments (Lee et al., 2018; de la Fuente-Nunez, 2019; Torres and de la Fuente-Nunez, 2019). Recent advances in computational biology have allowed the development of new molecular descriptors, which enable the discovery of potent AMP through exploitation of their vast sequence space (Awale et al., 2017; Lin et al., 2018). Genetic and pattern recognition algorithms are examples of successful tools that have been used for the generation of AMP antibiotics that display antimicrobial activity both *in vitro* and even in animal models (Lipkin and Lazaridis, 2017; Cipcigan et al., 2018; Pane et al., 2018; Pfeil et al., 2018; Porto et al., 2018; Rondon-Villarreal and Pinzon-Reyes, 2018). For example, Guavanin 2, an AMP generated by means of a genetic algorithm through a descriptive function that considered amphipathic distribution, net charge and hydrophobicity, was bactericidal at low concentrations, causing the disruption of *Pseudomonas aeruginosa* membranes by hyperpolarization of the membrane and displaying anti-infective activity in a mouse model (Porto et al., 2018).

*In vivo* studies in animals have demonstrated that AMP provide protection against microbial infection and that their absence results in an increased risk of infectious disease (Rivas-Santiago et al., 2009). In some cases, protection against infection is relatively generalised, i.e. effected by a number of AMP, such as the combination of drosomycin and metchnikowin and defence against *Candida albicans* infection in *Drosophila melanogaster* (Imler and Bulet, 2005; Hanson et al., 2019), whereas in others interactions are very specific, e.g., diptericin and defence against *Providencia rettgeri* infection in *D. melanogaster* (Hanson et al., 2019). Clinical correlations between AMP production and protection against infection exist that extend to humans (Hancock et al., 2016; Mangoni et al., 2016; de la Fuente-Nunez et al., 2017a; Coates et al., 2018), as patients with impaired epithelial AMP production (e.g., atopic dermatitis/eczema) are more susceptible to secondary infection, unlike those with increased AMP production (e.g., psoriasis) (Ong et al., 2002; Yamasaki and Gallo, 2008). Thus, it appears to be clear that AMP function as antimicrobials *in vivo*.

Despite the promise of AMP as novel antimicrobials, a lack of optimization and standardization of experimental conditions for antimicrobial susceptibility testing (AST), including exposure to different pH, salt solutions, serum half-life, and media/biological matrices used during AST (Mahlapuu et al., 2016; Torres et al., 2019) has been a major block to confirming efficacy potential from the outset in AMP drug development pathways. Standardization of experimental conditions for assessing the antimicrobial properties of AMP, and the difficulties encountered therein, are the subject of this manuscript. It is widely believed that AMP represent a group of molecules with the potential for development into a new generation of antimicrobials and for which “standard” AST protocols can significantly underestimate the AMP efficacy as antimicrobial drug candidates. In this era of increasing levels of AMR worldwide, drug development professionals cannot afford to ignore potential antimicrobial drug candidates simply because they do not perform well using “standard” laboratory test methods. Should AMP be successfully developed as therapeutics, due consideration needs to be given to manufacturing peptides on a large scale and safe and ethical disposal of manufacturing by-products and unused peptides. More than 60 peptide-based drugs have been already approved by the Food and Drug Administration (FDA) and more than 400 are in pre/clinical development (Aoki and Ueda, 2013; da Costa et al., 2015; Ageitos et al., 2016; Mahlapuu et al., 2016; Lee et al., 2019). Of these, at least 70 are AMP, with more than 25 in clinical trials (Koo and Seo, 2019). The peptide therapeutics market was valued at >$23 Bn (US) in 2017 and is predicted to be worth >$43 Bn (US) by 2024 (Zion Market Research, 2018). Additionally, peptide-based antimicrobials have been successfully used in the clinic for a number of years, including the antibacterials colistin, vancomycin, daptomycin and the antifungals of the echinocandin class (Hancock and Chapple, 1999; Mercer and O'Neil, 2013). The peptide components of the complex molecules are, in most cases, cyclic (head-to-tail cyclization) or restricted (side chain-to-side chain or side chain-to-end cyclization) or conjugated with other organic compounds, such as carbohydrates or lipids. Cyclization and/or conjugation confer AMP longer half-life and increased bioavailability, thus improving the probability of achieving a successful treatment (Greber and Dawgul, 2017).

## Antimicrobial Susceptibility Testing

Antimicrobial susceptibility testing (AST) determines the concentration of an antimicrobial that inhibits microbial growth, for both microbicidal and microbiostatic agents (Brown et al., 2016; Sanguinetti and Posteraro, 2018; Humphries et al., 2019; van Belkum et al., 2019). The importance of accurate AST in at least guiding antibiotic use in the clinic cannot be underestimated (Doern et al., 1994; Kumar et al., 2009; Weiss et al., 2012; Holmes et al., 2016).

During the development of novel antimicrobials, AST is vitally important; (i) to determine the preclinical activity of drug candidates and allow the identification of lead compounds, (ii) to facilitate the determination of the likelihood of resistance development, (iii) to provide estimates of likely *in vivo* and critically, clinical efficacy when testing compounds in biological matrices replicating sites of infection, e.g., blood/plasma/serum, lung bronchiolar lavage fluid/sputum, urine, biofilms, etc. (Breteler et al., 2011; Macia et al., 2014; Bottger et al., 2017; Ersoy et al., 2017; Nizet, 2017; Savini et al., 2017; Starr and Wimley, 2017; Haney et al., 2019).

## Antimicrobial Susceptibility Testing Methods for Existing Classes of Antimicrobials

Most AST, and its interpretation, is conducted using internationally recognised standards developed by bodies including the International Organization for Standardization (ISO), Clinical and Laboratory Standards Institute (CLSI), the European Committee on Antimicrobial Susceptibility Testing (EUCAST), The United States Committee on Antimicrobial Susceptibility Testing (USCAST) and the US Food and Drug Administration (FDA) Center for Drug Evaluation and Research (CDER) (Table 1) (Magiorakos et al., 2012; Kahlmeter, 2015; Humphries et al., 2019). Susceptibility Test Interpretive Criteria (STIC), also known as “breakpoints,” are used to determine the optimal dose of antimicrobials for treating infection and are based on those published by the CLSI, EUCAST, USCAST and the FDA. In December 2017 the FDA launched the Antimicrobial Susceptibility Test Interpretive Criteria website (https://www.fda.gov/drugs/development-resources/fda-recognised-antimicrobial-susceptibility-test-interpretive-criteria) which includes STIC similar to those published by CLSI and EUCAST. Different documents describe breakpoints for bacteria, yeasts, filamentous fungi (moulds) and other microorganisms (Table 1). Despite many similarities and agreements, there remains some lack of harmonisation between AST methods from different organisations (Pfaller et al., 2011, 2014; Chowdhary et al., 2015; Kahlmeter, 2015; Brown et al., 2016; Sanguinetti and Posteraro, 2018; Simjee et al., 2018; Cusack et al., 2019). Interpretive categories most commonly assigned are susceptible (S), indicative of a high probability of a successful outcome, and resistant (R), indicative of a low probability of a successful outcome, although in less common cases other categories include; non-susceptible, intermediate, susceptible-dose dependent and area of technical uncertainty (See the documents in Table 1 for details about these interpretive categories). An alternative STIC is the Epidemiological Cutoff Value (ECV CLSI, 2018f or ECOFF EUCAST, 2019c). The ECV/ECOFF is defined as the MIC that separates a population into isolates with and those without acquired or mutational resistance based on their phenotypic MIC value. An ECV is not a “breakpoint” as there is no clinical outcome or clinical trial data. Thus, an ECV is not a predictor of clinical success, but allows for prediction of whether an isolate has possible resistance to a given antimicrobial (Turnidge et al., 2006; Lockhart et al., 2017). For conventional antimicrobials with known resistance mechanisms, it is easier to define an ECV/ECOFF than a breakpoint, but for AMP, where resistance mechanisms are not necessarily known, or present, it is much more difficult (if not impossible) to define ECV/ECOFF, let alone a breakpoint. If that is the case, then defining STIC for AMP will require an entirely new definition.

**Table 1:** Internationally recognised standards for Antimicrobial Susceptibility Testing (AST) and Susceptibility Testing Interpretive Criteria (STIC)/Breakpoints.

**Organisation**	**Antimicrobial susceptibility testing document**	**Interpretive criteria document**	**References**
**Bacteria**			
CLSI	Methods for Dilution Antimicrobial Susceptibility Tests for Bacteria That Grow Aerobically. M07, ED11. Methods for Antimicrobial Susceptibility Testing of Anaerobic Bacteria. M11, ED9. Susceptibility Testing of Mycobacteria, *Nocardia* spp., and Other Aerobic Actinomycetes. M24, ED3. Methods for Antimicrobial Susceptibility Testing for Human Mycoplasmas. M43, ED1. Methods for Antimicrobial Dilution and Disk Susceptibility Testing of Infrequently Isolated or Fastidious Bacteria. M45, ED3.	Performance Standards for Antimicrobial Susceptibility Testing (M100 ED29) Performance Standards for Susceptibility Testing of Mycobacteria, *Nocardia* spp., and Other Aerobic Actinomycetes. M62, ED1.	CLSI, 2011, 2015, 2018a,b,c,d, 2019
EUCAST	Antimicrobial susceptibility testing: EUCAST disk diffusion method, Version 7.0. *EUCAST uses ISO 20776-1 for other bacterial AST methods*	The European Committee on Antimicrobial Susceptibility Testing. Breakpoint tables for interpretation of MICs and zone diameters. Version 9.0, 2019	EUCAST, 2019a,b
FDA		Antibacterial Susceptibility Test Interpretive Criteria, 2018	https://www.fda.gov/drugs/development-resources/antibacterial-susceptibility-test-interpretive-criteria
USCAST		2019 USCAST Interpretive tables	http://www.uscast.org/
ISO	Clinical laboratory testing and *in vitro* diagnostic test systems—Susceptibility testing of infectious agents and evaluation of performance of antimicrobial susceptibility test devices - Part 1: Reference method for testing the *in vitro* activity of antimicrobial agents against rapidly growing aerobic bacteria involved in infectious diseases. ISO20776-1.		ISO, 2019
**Yeasts**			
CLSI	Reference Method for Broth Dilution Antifungal Susceptibility Testing of Yeasts. M27, ED4. Method for Antifungal Disk Diffusion Susceptibility Testing of Yeasts. M44, ED3.	Performance Standards for Antifungal Susceptibility Testing of Yeasts, M60, S1. Epidemiological Cutoff Values for Antifungal Susceptibility Testing, M59, ED2.	CLSI, 2017a,b, 2018e,f
EUCAST	Method for the determination of broth dilution minimum inhibitory concentrations of antifungal agents for yeasts. E.DEF 7.3.1.	The European Committee on Antimicrobial Susceptibility Testing: Breakpoint tables for interpretation of MICs. Version 9.0, 2018	EUCAST, 2017a, 2018
**Filamentous Fungi**			
CLSI	Reference Method for Broth Dilution Antifungal Susceptibility Testing of Filamentous Fungi. M38, ED3. Method for Antifungal Disk Diffusion Susceptibility Testing of Nondermatophyte Filamentous Fungi. M51, ED1.	Performance Standards for Antifungal Susceptibility Testing of Filamentous Fungi. M61, ED1. Epidemiological Cutoff Values for Antifungal Susceptibility Testing, M59, ED2.	CLSI, 2010, 2017c,d, 2018f
EUCAST	Method for the determination of broth dilution minimum inhibitory concentrations of antifungal agents for conidia forming moulds. E.DEF 9.3.1.	The European Committee on Antimicrobial Susceptibility Testing: Breakpoint tables for interpretation of MICs. Version 9.0, 2018	EUCAST, 2017b, 2018

The most commonly used manual AST methods are disk diffusion and broth microdilution, although many large hospital laboratories use automated systems, due to improvements in convenience and flexibility, such as BD Phoenix™, Beckman Coulter MicroScan, bioMerieux Vitek® 2, Accelerate Diagnostics PhenoTest and Thermo Fisher Sensititre™ (Syal et al., 2017). In the case of antifungal susceptibility testing, broth microdilution is almost exclusively used (Pfaller and Diekema, 2012; Ostrosky-Zeichner and Andes, 2017). The time taken for conventional AST varies considerably, depending on the infectious agent, and normally is only performed after the pathogen has been cultured and identified at the species level (van Belkum et al., 2019). For bacteria this can be as quick as 24–48 h, but for fungi, isolation and identification can take days, if not weeks, rather than hours and AST may take 48 h or longer (CLSI, 2012, 2017a,b; EUCAST, 2017a,b). Any delay in appropriate antimicrobial therapy can lead to increased mortality for severe infections (Delaloye and Calandra, 2014; Liu et al., 2017). There is, therefore, an urgent need for more rapid AST (Sanguinetti and Posteraro, 2017; Kim et al., 2018; Cansizoglu et al., 2019; Idelevich and Becker, 2019).

The standards for AST available from ISO, EUCAST and CLSI were first implemented almost 60 years ago (World Health Organization, 1961) and have remained largely unchanged since then in a “one size fits all” approach and are used largely unquestioningly by many users (Nizet, 2017). However, some antimicrobials (and microorganisms) do not work in these standards and require modifications to the testing procedures, either by the use of additives to standard media to generate representative efficacy values or by the use of alternative or modified media for fastidious microorganisms (e.g., Haemophilus Test Medium for *Haemophilus influenzae* and *H. parainfluenzae* and the addition of 2.5–5.0% (v/v) lysed horse blood to cation-adjusted Mueller-Hinton (CA-MH) medium when testing streptococci) (CLSI, 2018a,b). Interestingly, EUCAST developed a different medium for use with fastidious bacteria (including streptococci and *Haemophilus* spp.; a modified version of MH agar, with the addition of 5% mechanically defibrinated horse blood and 20 mg/L β-nicotinamide adenine dinucleotide (β-NAD) (Matuschek et al., 2018). Although most efficacy end-points are 100% growth inhibition, there may be a lesser burden for some pathogen/antimicrobial combinations (e.g., ≥50% growth inhibition for fluconazole, flucytosine and ketoconazole for non-dermatophyte moulds CLSI, 2017c) or the determination of minimum effective concentrations (MEC), rather than MIC (e.g., the MEC of echinocandins vs. filamentous fungi is defined as “the lowest concentration of an antifungal agent that leads to the growth of small, rounded, compact hyphal forms compared with the hyphal growth seen in the control well” CLSI, 2017c). Consideration of other assay parameters, such as those described in Table 2, perhaps require attention when conducting these standard procedures or when they are updated.

**Table 2:** Factors influencing antimicrobial activity of AMP.

***In vitro***	***Ex vivo***	***In vivo***
pH and ionic strength	Biological matrices (e.g., blood)	Animal models of infection
Temperature	Mammalian cells	Pharmacokinetics
Medium type/composition	Intracellular pathogens	Pharmacodynamics
Nutrient concentrations	Air:Liquid or Solid:liquid interface	Metabolic interactions
Buffer	Infection models	Polypharmacy (drug- drug interactions)
Bicarbonate		Formulation and delivery
Metal ions		Polymicrobial infections
Salt (NaCl)		
Polysorbate-80		
Synergy/Antagonism with other antimicrobials		
Inoculum size		
Growth Phase (e.g., biofilms, persisters, spores, small colony variants, and other phenotypic variants)		
Charge effects		
Solubility		
Laboratory materials		
Proteolysis		
Biological macromolecules (e.g., protein, DNA)		
Oxygen (hyper-, norm- and hypoxia)		
Mono/Polymicrobial interactions		

*In the context of this manuscript, ex vivo refers to experiment parameters that are not in a living host organism (out of the living), but are simulating in vivo conditions. In vivo refers to experiments conducted in/on a living host organism*.

When using the same AST method, e.g., broth microdilution, results can be influenced by factors such as medium age, presence of polysorbate 80 and ion content (Bradford et al., 2005; Fernandez-Mazarrasa et al., 2009; Sader et al., 2012; Sutherland and Nicolau, 2014) as can non-compliance with AST standards (Mouton et al., 2018; Turner and Ashley, 2019). Examples where modifications to existing AST methods have been successfully implemented include the lipopeptide antibiotic daptomycin. Daptomycin requires physiological concentrations of calcium (50 mg/L) in the medium for optimal efficacy or otherwise MIC values can be up to 32-fold higher, clearly affecting whether an isolate could be sensitive or resistant (Eliopoulos et al., 1986; Campeau et al., 2018) and therefore the CA-MH broth or agar is supplemented with additional Ca^2+^ (CLSI, 2018a,b). The lipoglycopeptides antibiotics (including oritavancin, dalbavancin, and teicoplanin) are subject to binding to laboratory plasticware (Arhin et al., 2008; Ross et al., 2014) and therefore CLSI recommends addition of 0.002% (v/v) polysorbate 80 (Tween 80) to CA-MHB to prevent such binding (CLSI, 2018a). When testing staphylococci for sensitivity to oxacillin (MRSA phenotype) it is recommended to supplement media with 2% (w/v) sodium chloride as this enhances the expression of mecA-mediated oxacillin resistance and reduces the reporting of false negatives that occurs when 5% (w/v) sodium chloride is used (Huang et al., 1993; Brown, 2001). Additionally, CLSI recommends that when testing for oxacillin resistance in staphylococci samples should be incubated at 33–35°C, as testing at temperatures above 35°C may not detect *mecA*-mediated resistance (CLSI, 2018a). Therefore, antimicrobial substance-specific changes can be made to “standard” AST methods, so there is no reason why this should not be possible for AMP in pre-/clinical development. Additionally, AMP-specific end points do not necessarily have to equate to 100% growth inhibition. Obviously, any and all deviations from “standard” protocols will require rigorous and detailed justification and validation.

AMP represent one such group of molecules for which these “standard” protocols can significantly underestimate their efficacy potential. In this era of increasing levels of AMR worldwide, can drug developers really afford to ignore potential antimicrobial drug candidates simply because they do not perform well using “standard” test methods? AST of AMP is normally performed using the broth microdilution (or macrodilution) procedure, as many AMP are positively charged molecules (and are dependent on that positive charge for activity Jiang et al., 2009; Mercer et al., 2019; Torres et al., 2019). Positively charged AMP interact with negatively charged components in agar and neutralising their activity, meaning that disk diffusion AST methods significantly under-estimate activity or mask it completely (Kunin and Edmondson, 1968; Lehrer et al., 1991), as is the case for the peptide antibiotics daptomycin (Humphries et al., 2013) and colistin (Albur et al., 2014; Poirel et al., 2017; Matuschek et al., 2018).

If AMP are to realise their potential as a future generation of anti-infective therapeutics, AST methods will require approval from regulatory authorities and buy-in from organisations such as EUCAST, CLSI, and ISO (Kahlmeter, 2015; CLSI, 2018g). To do this, any AST method for AMP or modification to an AST method, at least for EUCAST, must be calibrated to the ISO broth microdilution technique, often as part of the formal accreditation process (Kahlmeter, 2015; ISO, 2019). Therefore, any AST method must be accurate, robust, reproducible, have clinical utility and validity and, ultimately, be amenable to automation.

## Antimicrobial Susceptibility Testing of AMP

It is clear that current AST methodologies are not “fit-for-purpose” for determining the activity of AMP *in vitro*. When considering a single antimicrobial and a single species of microorganism, inter-laboratory variation and biological variation (i.e., variation between strains) can still cause a broad distribution of wild-type MIC values (Annis and Craig, 2005; Hombach et al., 2016; Mouton et al., 2019). Therefore, efforts to introduce standardised AST methods for AMP may turn out to be an extremely onerous and difficult task (Jepson et al., 2016). Previous attempts to standardise AST for AMP have met with limited success. The method devised by the Hancock lab (http://cmdr.ubc.ca/bobh/method/modified-mic-method-for-cationic-antimicrobial-peptides/), was an adaptation of the CLSI broth microdilution procedure (CLSI, 2018a), but has not been widely adopted. The main differences between the two methods were careful choice of labware (polypropylene or Sigmacote-coated glass), diluent used (0.01% (v/v) acetic acid containing 0.2% (w/v) bovine serum albumin) and determining an MIC_50_ (reduction in growth of ≥50%) in the Hancock method. A comparison of the two methods revealed differences in MIC in which the Hancock method generated MIC values that were predominantly lower than the CLSI method (Giacometti et al., 2000). In the two decades since the publication of this method, interest in AMP has grown significantly, so consideration of AST for AMP is perhaps overdue a review and update.

**Figure 1:**
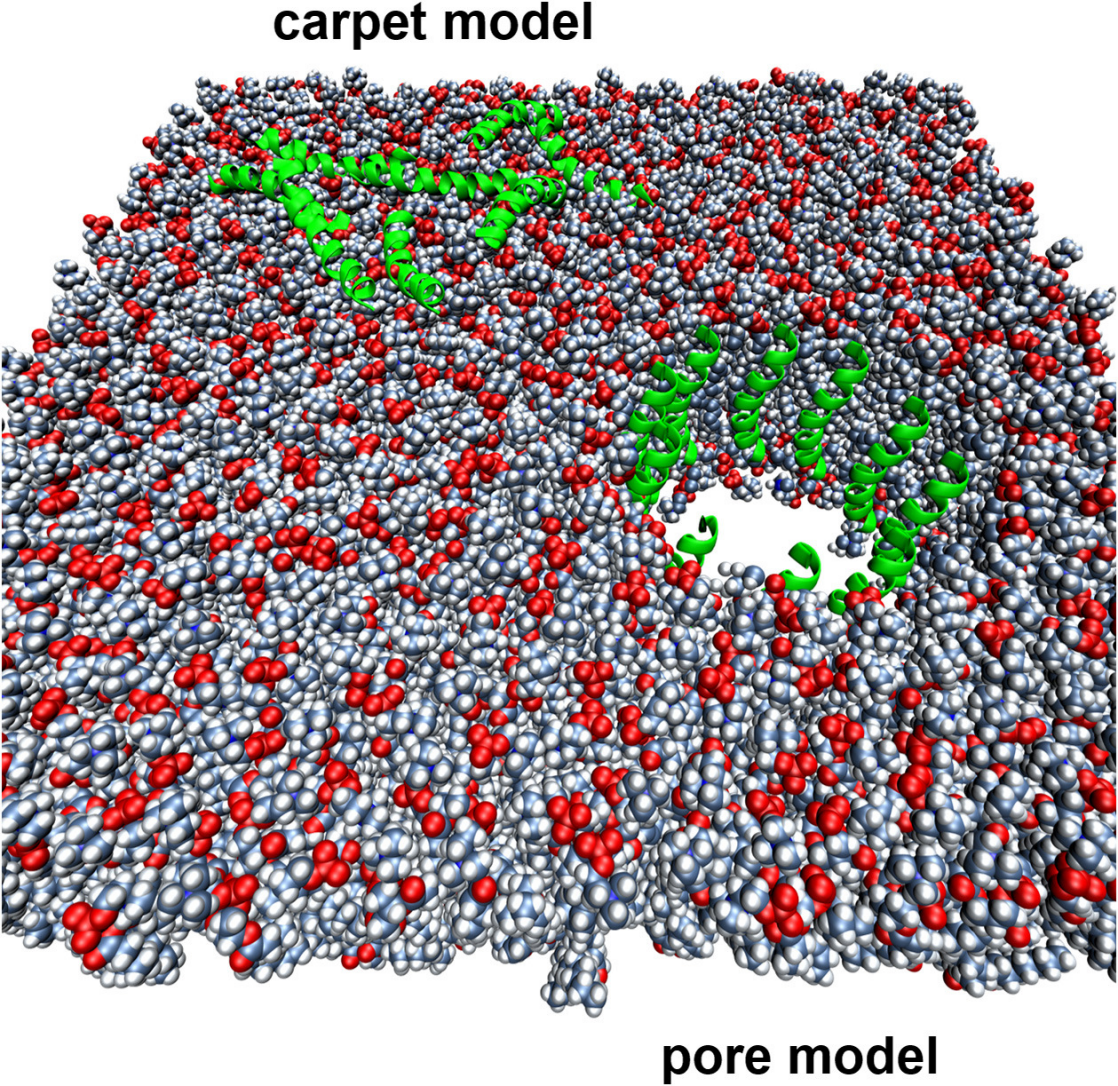
Two different mechanisms of action proposed for membrane-active AMPs. The lipid bilayer is represented by spheres, while the AMPs are represented by green helical cartoons.

Membrane-active AMP are typically associated with higher MIC values than those of conventional antibiotics, although this is not always the case. Is this simply because the membrane represents a very large target, especially for AMP that kill via the carpet mechanism and even some pore-formers (Figure 1), or is this because currently used test methods are not fit for purpose? For example, Roversi et al. determined that for the cathelicidin peptide PMAP-23 to kill *E. coli* ATCC25922 required 10^6^-10^7^ bound peptides per cell (1–10 μM; 2.7–27 mg/L) (Roversi et al., 2014). This compares to a typical susceptible MIC distribution of ciprofloxacin vs. *E. coli* of 0.004–0.064 mg/L (0.012–0.193 μM) (*R* > 0.5 mg/L; ECOFF = 0.064 mg/L) (http://www.eucast.org) and which specifically targets the enzymes DNA gyrase and topoisomerase IV, thereby preventing DNA replication. PMAP-23 kills bacteria by the “carpet” model (Orioni et al., 2009), so this is likely to be more peptide molecules than required for pore-forming peptides, or those with more specific mechanisms of action (Figure 1).

A lack of antimicrobial activity of AMP, or attenuation of activity, has been observed for AMP when assayed under conditions in which existing, conventional antibiotic classes are active. This has been ascribed to a variety of features, including inactivation by physiological concentrations of NaCl and divalent metal cations or serum/plasma. However, manipulation of media conditions, including making them more physiologically relevant, could potentially reverse the inhibitory effects of compounds such as NaCl and proteins. For example, addition of sodium bicarbonate was able to reverse the inhibitory effects of physiological NaCl concentration against a number of different AMP, including the human cathelicidin LL-37, human β-defensin 2 (hBD-2) and Cryptidin-4, albeit not dermcidin (Dorschner et al., 2006). Many of the factors for consideration described here have also been investigated for their effects on the activity of conventional antimicrobials with varying outcomes (Hajdu et al., 2010; Erdogan-Yildirim et al., 2011; Ersoy et al., 2017; Oesterreicher et al., 2019).

It has been suggested that the discovery and preclinical development of new antimicrobials should target pathogens as they are found at sites of infection, rather than the potentially different phenotype demonstrated in microbiological growth media (Dorschner et al., 2006; Ersoy et al., 2017). One reason for this is because standard AST does not take into account the potential influence (positive or negative) of the host cell environment on microbial susceptibility and resistance (Sutherland and Nicolau, 2014; Haney et al., 2019). In order to create a standardised AST procedure suitable for AMP there are a number of factors that will need to be considered (Table 2).

Whilst many of the factors described in Table 2 may have an impact on the design of AST for AMP, or the MIC of individual AMP, combinations of changes to these factors must also be considered (Oesterreicher et al., 2019).

### Laboratory Materials

A number of publications have demonstrated that the results of AST of many antimicrobials, not just AMP, can be affected by the choice of laboratory plasticware for use with the broth microdilution procedure and even the choice of tubes used for preparing reagents (Singhal et al., 2018; Kavanagh et al., 2019). This also applies to AST of AMP (Otvos and Cudic, 2007; Wiegand et al., 2008; Kristensen et al., 2015).

Some peptide-based antibiotics and AMP need to be prepared in alternative solvents, or with additives included in the media/diluent to prevent binding to the surfaces of tubes and plates. The lipoglycopeptides, including oritavancin and telavancin, must be solubilised in dimethyl sulfoxide (DMSO) or with the addition of a surfactant, 0.002% (v/v) polysorbate 80 (Tween 80), added to the water to prevent binding (Arhin et al., 2008; Ross et al., 2014). When DMSO was used in place of water as solvent for AST of the echinocandins (lipopeptide antifungals) caspofungin and micafungin, MIC values were lower and MIC ranges were narrower (Alastruey-Izquierdo et al., 2012) as was the case when water was supplemented with 50% bovine serum albumin (Arendrup et al., 2011; Garcia-Effron et al., 2011). In contrast, even though colistin (cyclic lipopeptide) binds to plastics (Karvanen et al., 2017), the CLSI and EUCAST recommend broth microdilution for AST of colistin, but without added surfactant (Hindler and Humphries, 2013; CLSI-EUCAST, 2016), as polysorbate 80 can act synergistically with polymyxins and reduce MICs (Brown and Winsley, 1968; Ezadi et al., 2019), presumably due to interactions with lipopolysaccharides in the Gram negative outer membrane (Correa et al., 2017). Additionally, polysorbate has antibacterial properties of its own (Brown et al., 1979; Figura et al., 2012). In the case of AMP, the potential use of polysorbate is more complex as the addition of polysorbate 20 improved antiviral activity of LL-37 and magainin-2B amide, but reduced antibacterial activity (Ulaeto et al., 2016), however the impact of polysorbate on AMP activity has not been extensively studied. The effect of polysorbate for the prevention of binding of AMP to laboratory plastics would need to be carefully investigated in light of possible synergistic activity as observed with the cationic, membrane-active polymyxins. This is clearly an area that requires more detailed investigation before any recommendation specific to AMP susceptibility testing can be made. In the method described by the Hancock laboratory (http://cmdr.ubc.ca/bobh/method/modified-mic-method-for-cationic-antimicrobial-peptides/) for AST of AMP, they recommend the use of a diluent of 0.01% acetic acid containing 0.2% bovine serum albumin (BSA) to reduce peptide binding to plastic surfaces (Wiegand et al., 2008), although it is difficult to ascertain whether this recommendation has been broadly adopted. Even when it comes to the choice of filter for filter-sterilisation of AMP-containing solutions, caution may be required, as cationic AMP may bind to negatively charged membranes, such as cellulose acetate (Wiegand et al., 2008).

The choice of laboratory plasticware is similarly problematic as there are a number of publications that indicate that cationic AMP bind to polypropylene, polystyrene and borosilicate glass, the most commonly used materials used in the manufacture of labware (Chico et al., 2003; Kristensen et al., 2015), although the extent of binding may be similar regardless of the type of material used (Sanchez-Gomez et al., 2008; Kristensen et al., 2015). This situation also applies to some antibiotics (see above) and even antifungals such as the echinocandins (Fothergill et al., 2016; Arendrup et al., 2019). In the study of Kristensen and co-workers, binding to the surface of the tubes (irrespective of material) was saturable and relatively more peptide bound at low concentrations, so a simple solution may be to keep peptide concentrations as high as possible to minimise the percentage lost to protein binding. Irrespective of this, this would lead to an underestimation of AMP efficacy when trying to determine MIC values at lower peptide concentrations. The use of low protein-binding plasticware demonstrated reduced binding of selected AMP when compared to polystyrene, polypropylene and borosilicate glass (Kristensen et al., 2015), but they unfortunately did not carry out AST in this study, so it is not currently possible to assess whether the use of low protein-binding labware would lead to a reduction in MIC for AMP without further studies and, additionally, this is unlikely to provide a low cost solution.

The possibility of using different diluents, additives, or changes to laboratory plasticware clearly warrants further investigation, but perhaps the best recommendations are to be as consistent as possible with your choices of materials and diluents and keep AMP concentrations high where possible to minimise the relative amount of peptide loss by binding.

### Media Composition

Most AST using AMP is conducted using broth dilution methods described by EUCAST or CLSI, or close variants thereof. For bacteria, when conducting broth dilution AST, both organisations recommend the use of Mueller-Hinton broth (MHB) containing 10–12.5 mg/L Mg^2+^ and 20–25 mg/L Ca^2+^ (CLSI, 2018a; EUCAST, 2019a), whereas for testing of fungi, both recommend RMPI-1640 medium (RPMI), but with different concentrations of glucose; 2.0 g/L for CLSI test methods and 20.0 g/L for EUCAST test methods (CLSI, 2017a,c; EUCAST, 2017a,b). The reasons for the almost universal adoption of MHB for antibacterial susceptibility testing are unclear, but MHB serves as a poor simulation of normal human body fluids (Ersoy et al., 2017; Nizet, 2017). Antifungal susceptibility testing using RPMI-1640 liquid medium (CLSI, 2017a,c; EUCAST, 2017a,b) more closely simulates normal human body fluids, whereas CLSI disk diffusion testing of fungi and yeasts again uses Mueller-Hinton agar (MHA) (CLSI, 2010, 2018e). The effect of media type on AST results has been examined in a number of studies (Schwab et al., 1999; Sajjan et al., 2001; Dorschner et al., 2006; Kumaraswamy et al., 2016; Ersoy et al., 2017; Tucker et al., 2018). The use of MHB for AST of cationic AMP may be an unsuitable medium due to the high content of anionic amino acids in hydrolysed casein. MHB contains 17.5 g/L acid hydrolysate of casein, with only 3 g/L beef extract and 1.5 g/L starch, as well as additional Ca^2+^ (20–25 mg/L final concentration) and Mg^2+^ (10–12.5 mg/L final concentration). Casein contains 25% anionic amino acids and these can interfere with cationic AMP activity and cause them to precipitate (Turner et al., 1998). Turner and co-workers compared the activity of LL-37 and protegrin (PG-1) in conventional MHB and MHB that had first been passed through an anion exchange column to deplete the MHB of anionic compounds and demonstrated that MICs against *E. coli* ML-35p, *P. aeruginosa* MR3007, *Bacillus subtilis* and *S. aureus* 930918-3 were 3 - >20-fold higher in standard MHB compared to MHB passed through an anion exchange column (Turner et al., 1998). The choice of media can make a significant impact on AMP efficacy. Incubation for 2 h with 5 μM of the cationic AMP D4E1 resulted in 100% killing of ~1 × 10^5^ cfu of *S. aureus* when incubated in RPMI-1640 liquid medium buffered with 30 mM HEPES, whereas the use of MHB, CA-MHB, nutrient broth + 125 mM NaCl or Tryptone soya broth (TSB) resulted in less killing (68.6, 22.2, 56.1, and 5.3%, respectively). Similar trends were observed for *P. aeruginosa* ATCC27853 and another cationic AMP (D2A21) (Schwab et al., 1999). In the Schwab study, a range of different buffers were also tested (PBS, Tris-NaCl, citrate-phosphate, saline and phosphate), with a trend for less killing in the phosphate buffers. Interestingly, osmolarity did not have any effect on the activity of D2A21 against a range of bacterial pathogens isolated from patients with cystic fibrosis (CF) (Schwab et al., 1999). When determining the efficacy of the histatin derivative P-113, Sajjan and co-workers observed little activity in CA-MHB, but when this was diluted 20-fold the MIC was 3.1 mg/L, indicating that one or more components of CA-MHB were inhibitory to the antimicrobial activity of P-113 against *P. aeruginosa*. Use of an alternative medium (LM broth) that attained similar growth and growth rates to CA-MHB, but with reduced concentrations of monovalent and divalent salts, retained the efficacy of P-113 to a range of bacterial CF pathogens, but did not affect the efficacy of tobramycin, ceftazidime or imipenem, indicating the detrimental effect of monovalent and/or divalent salts on AMP efficacy (Sajjan et al., 2001).

The activity of azithromycin against different isolates of *Stenotrophomonas maltophilia* was examined in cation-adjusted MHB (CA-MHB) and RPMI-1640 liquid medium supplemented with 10% Luria-Bertani broth (LB) (Kumaraswamy et al., 2016). In this study MICs in MHB were 32–256 mg/L, whereas in RPMI, MIC values were significantly lower (0.03–0.25 mg/L) and azithromycin also synergised with the AMP LL-37 against MDR *P. aeruginosa* and MDR *Acinetobacter baumannii*. When tested *in vivo*, azithromycin also resulted in clearance of *A. baumannii* and *S. maltophilia*, sensitising *S. maltophilia* to neutrophil killing (Lin et al., 2015; Kumaraswamy et al., *2016)*. A series of 20 AMP were isolated from a surface-displayed peptide library and tested for activity in MHB via broth microdilution, but only 2 of the peptides, and cecropin P1, demonstrated an MIC, but when the MBC was determined in 10 mM Tris, pH 7.4 + 25 mM NaCl, all AMP were bactericidal (MBC ≤ 128 μM) against at least 1 of 4 Gram negative bacteria (Tucker et al., 2018). In other cases, the use of diluted nutrient media results in improved efficacy of AMP in AST. The MIC of the lactoferricin derivative HLopt2 against selected *Candida* spp. was >250 mg/L, but when tested in BHI diluted 1:100 the minimum microbicidal concentrations (MMC) were 2–31 mg/L. Similarly, the MIC in CA-MH vs. *P. aeruginosa* and *S. aureus* was much higher (125 and 63 mg/L, respectively) than the MMC when tested in 1:100-diluted BHI (4 and 2 mg/L, respectively) (Ptaszynska et al., 2019). In some cases, the choice of media type can have detrimental effects on AMP activity. For example, the semi-synthetic AMP Lin-SB056-1 was bactericidal against 6 *P. aeruginosa* in 1% TSB (MBC = 1.56–3.12 mg/L), whereas activity was lost in 80% artificial sputum medium, closely resembling the CF lung, except in the presence of additional EDTA (ethylenediaminetetraacetic acid), where bactericidal activity was restored (Maisetta et al., 2017).

The use of a more physiologically representative cell culture medium (RPMI-1640) may be better suited for AST of AMP, and is certainly more representative of conditions *in vivo* than CA-MHB or other nutrient-rich microbiological growth media and that the presence of higher concentrations of anionic substances adversely affects the efficacy of cationic AMP. In a caveat to this, it has recently been observed that the composition of a number of cell culture media, including RPMI-1640, do not simulate bodily fluids particularly closely (McKee and Komarova, 2017), so perhaps further work is required to develop media that more closely simulates mammalian bodily fluids.

### Solubility and Aggregation

Given that most AMP have a net positive charge and hydrophilic regions, aqueous solubility at concentrations required for AST are not problematic, although some AMP aggregate and become insoluble at relatively high concentrations in certain media and in the presence of selected anions. This can result in a loss of activity, stability and/or increased cytotoxicity (Frokjaer and Otzen, 2005; Ratanji et al., 2014; Haney et al., 2017). Hydrophobic regions in peptides are known to self-associate and drive the formation of aggregates (Kim and Hecht, 2006), so modifications to AMP design may reduce/prevent aggregation (Haney et al., 2017). For example, the AMP Temporin L forms aggregates in water and due to an extended hydrophobic region, but substitution of glutamine to lysine at position 3 significantly reduced aggregation in water and improved its antiendotoxin properties (Srivastava and Ghosh, 2013), whereas substitution with arginine at the same position improved activity against *P. aeruginosa* (and is also likely to attenuate aggregation) (Mangoni et al., 2011). For example, significant aggregation of the immunomodulatory AMP IDR-1018 occurred in the presence of phosphate, benzoate, nitrate, and citrate, but less aggregation was observed in the presence of acetate, chloride, water and, perhaps significantly, bicarbonate (see later). IDR-1018 also exhibited aggregation in 10% RPMI and 1% MEM tissue culture media and also co-precipitated with serum proteins in a concentration-dependent manner, in many cases adversely affected the desired immunomodulatory properties of the peptide and increased cytotoxicity (Hartlieb et al., 2017). Protegrin-4 (PG-4) also aggregates in the presence of phosphate (>2.0 mg/ml PG-4 in 50 mM sodium phosphate buffer, pH 7.4), forming amyloid-like fibrils and retained antimicrobial activity against *B. subtilis* (Gour et al., 2019). LL-37 exists in equilibrium between monomers and oligomers in solution at low concentrations and oligomerizes in the presence of zwitterionic membranes (Johansson et al., 1998; Oren et al., 1999). Dermaseptin S9 formed aggregates and amyloid-like fibrils and the peptide binds to membranes in an aggregated state (Caillon et al., 2013). Human α-defensin 6 (HD6) is a 32-residue cysteine-rich peptide that lacks the broad-spectrum antimicrobial activity observed for other human α-defensins. Strikingly, HD6 oligomerises to form “nanonets,” due to the disposition of hydrophobic residues in the HD6 primary structure, that entrap microbes and prevent invasive pathogens such as *Salmonella enterica* serovar Typhimurium and *Listeria monocytogenes* from entering host cells in the gastrointestinal tract (Chu et al., 2012; Chairatana and Nolan, 2017). An *in silico* analysis of the aggregative potential of AMP and non-antimicrobial peptides revealed that AMP demonstrate very low *in vitro* aggregation propensity, but high *in vivo* aggregation propensity. Non-antimicrobial peptides can be divided in two main groups, presenting either high or low values for both *in vivo* and *in vitro* aggregation. These results suggest that most AMP demonstrate minimal aggregation in aqueous solution, but promote aggregation in a more hydrophobic environment (i.e., the bacteria cell membrane) (Torrent et al., 2011), something borne out in many experimental studies. Thus, when conducting AST with AMP, consideration of the solute/s used can be important and, in most cases, it would be advantageous to use aqueous solutions where possible for formulation of drugs for human or animal use, to minimise aggregation and to carefully assess peptide solubility in the media used for AST.

### Biological Matrices

When testing the efficacy of any antimicrobial, logic dictates that it would be practical to test efficacy in the biological matrix at the site of infection, e.g., blood, sputum, urine, etc., but this is unlikely to be practical for routine screening. However, prior to the initiation of *in vivo* efficacy studies it would be sensible to determine the efficacy of AMP in relevant biological matrices. Proteolytic degradation of AMP is often considered a major weakness limiting their potential therapeutic application, as is binding to biological matrices, including serum/plasma proteins (Wang et al., 1998; Sivertsen et al., 2014), nucleic acids (Park et al., 1998; Hsu et al., 2005), ribosomes (Mardirossian et al., 2014) other proteins (Tu et al., 2011) bacterial cell walls (Malanovic and Lohner, 2016) and lipopolysaccharide (Piers et al., 1994; Sun and Shang, 2015). Many AMP can also bind to host cells as part of their innate immune system functions (van der Does et al., 2019) that are present in most biological matrices. For example, LL-37 has direct interactions with ≥16 proteins/receptors, that subsequently interact with >1000 secondary effector proteins and when used to stimulate monocytes >900 gene expression changes were observed (Hancock et al., 2016). It is, therefore, not unreasonable to assume that many AMP will interact with host cells and that the effects of these interactions may not necessarily be desirable. This may not need to be assessed when considering *in vitro* AST, but would need consideration, for example, at later stages of the drug development process. Significant efforts have been made to improve AMP activity in blood, plasma and/or serum (Hamamoto et al., 2002; Knappe et al., 2010; Nguyen et al., 2010; Chu et al., 2013; Dong et al., 2018; Kumar et al., 2018). When considering AMP activity in blood, serum or plasma the source of the blood and the method of collection must be taken into account. Most blood samples are collected in tubes (vacutainers) containing an anticoagulant to prevent blood clotting and it is known that the presence of an anticoagulant can affect AMP activity. For example, EDTA and citrate are known to enhance the efficacy of some AMP as they can chelate divalent cations which are inhibitory to the activity of a number of AMP (Wei and Bobek, 2005; Walkenhorst et al., 2014; Maisetta et al., 2017; Umerska et al., 2018; Grassi et al., 2019) and EDTA and heparin can inactivate proteases found in blood, including metalloproteases (EDTA), thrombin and Factor Xa (heparin) that may prevent AMP hydrolysis (Bowen and Remaley, 2014; Bottger et al., 2017; Rawlings et al., 2018).

The stability of proline-rich AMP (apidaecin and oncocin derivatives) was examined in murine blood, serum and plasma and, perhaps surprisingly, the general trend was for greatest stability in whole blood, followed by plasma and least stable in serum, albeit only over a 1 h incubation period, and that substitution of L-arginine residues for D-arginine or ornithine improved stability in blood, serum and plasma (Bottger et al., 2017). In another study, pre-incubation of a panel of AMP with red blood cells (RBC) (1 × 10^9^ RBC/ml) before exposure to the pathogen significantly increased the MIC of most AMP, with a similar inhibitory effect caused by serum, even though the affinity of AMP for bacteria was much greater than for RBC. Thus, serum binding and binding to host cells for AMP intended for systemic delivery requires consideration and can be adapted to AST testing in the presence of serum or host cells (Starr et al., 2016). However, when the AMP DNS-PMAP23 or esculentin-1a(1-21)NH2 was added directly to a mixture of RBC and *E. coli*, no significant inhibition of antibacterial activity took place (Savini et al., 2017), indicating that experimental set-up, and by extension the nature of the infection being potentially treated, is an important consideration. In another study, in the absence of host cells, WLBU2 (12.5 μM) retained activity in the presence of 98% human serum, whereas LL-37 was not active at concentrations up to 100 μM (Deslouches et al., 2005) and therefore the effect of biological matrices on AMP activity may be peptide-specific.

The efficacy of the histatin-derived AMP, P-113, was tested in diluted sputum from CF patients and no activity vs. *P. aeruginosa* was observed. When the stability of P-113 was determined in sputum, half-lives of 2.8–58.5 min were determined, probably preventing activity in sputum. By switching the composition of P-113 from all L-enantiomer amino acids to all D-enantiomer amino acids (P-113d), the activity against *P. aeruginosa* was comparable to the L-enantiomer peptide, but P-113d was stable in CF sputum for 7 d. When the efficacy of P-113d was tested in CF sputum, an additional 1 log kill of *P. aeruginosa* was attained in 1 h and efficacy was enhanced further by pre-treatment of the sputum with recombinant human deoxyribonuclease (rhDNase; Pulmozyme®), a therapeutic used in some CF patients (Sajjan et al., 2001). Interestingly, the activity of an all L-isomer of the AMP temporin lost activity in faeces within 30 min, whereas the all D-isomer version retained activity for 30 min, but activity was lost after 24 h (Oh et al., 2000). Thus, testing of AMP in biological matrices can be factored into AST and the earlier this is conducted will have a bearing on lead selection for AMP intended for specific infections.

### pH and Ionic Strength

More than 30 AMP are known to have pH-dependent activity, including LL-37, histatins, psoriasin, and lactoferrin, with greater activity predominantly observed at lower pH values, especially for histidine-containing AMP such as clavanins (Lee et al., 1997; Malik et al., 2016; Alvares et al., 2017). Changes in pH can affect ionic interactions between membranes and AMP by changing the protonation states of functional groups on the membrane and/or AMP, as well as effects on the ionic strength of the solution (Walkenhorst, 2016). Cationic AMP are normally more efficacious at neutral and lower pH due to the loss of net positive charge at alkaline pH (Malik et al., 2016). For example, the efficacy of LL-37 against *C. albicans* was greater at pH 4.5 (81% death) when compared to pH 5.5 (79% death) and pH 7.2 (40% death) (Lopez-Garcia et al., 2005). Localised pH can significantly impact the interaction of AMP with membranes and their subsequent ability to perturb the membrane (Malik et al., 2016; Alvares et al., 2017). This can reflect their predominant site of action, e.g., skin. The ionic strength of the buffer can also influence efficacy, with reduced efficacy often observed at higher ionic strengths (or an over-estimation of activity at low ionic strengths) (Sanchez-Gomez et al., 2008; Walkenhorst et al., 2013). Additionally, the choice of buffer can affect AMP efficacy, with greater efficacy observed in MOPS compared to phosphate buffer at similar pH and ionic strength (Walkenhorst, 2016). However, there are a number of exceptions to this, that may be peptide-dependent or organism dependent. Walkenhorst and co-workers observed the expected trend of enhanced activity at lower pH values for a family of peptides versus Gram negative bacteria and *C. albicans*, but the opposite effect was observed against *S. aureus*, with enhanced efficacy at higher pH values and hypothesized that this was due to changes to teichoic acids in the *S. aureus* cell wall, making the peptidoglycan layer less negatively charged at neutral and acidic pH (Walkenhorst et al., 2013). Interestingly, a linear form of esculentin 2EM caused greater cell lysis at pH 8.0 compared to pH 6.0 and this correlated with increased α-helicity of the peptide (Malik et al., 2016), indicating that the effect of pH on AMP activity cannot be readily predicted and needs to be determined where necessary.

Despite the effect of pH on AMP activity (which in many cases has not been investigated), AST of AMP is predominantly carried out at neutral pH and the effect of pH modulation is not considered. When considering the relevance of pH on AST of AMP, the main consideration should be of the pH at the site of infection and during delivery to the site of infection. Many assume that this is close to neutral, as an often cited physiological pH is 7.4, although pH can range from 7.0 to 9.0 in blood (Kellum, 2000). However, physiological pH values cover a relatively broad range, including pH 5.0 in the macrophage phagosome a site where intracellular pathogens such as *K. pneumoniae, Salmonella typhimurium, E. coli* and *Mycobacterium tuberculosis* can reside (Underhill and Ozinsky, 2002) and between 4 and 7 on the skin surface, with a most frequently determined pH range of 4.0–5.9 (Lambers et al., 2006), although the pH of chronic wounds can be alkaline (pH 7.15–8.9) (Gethin, 2007). Skin infection can cause an elevation in skin pH, as can other conditions, such as diabetes mellitus, that can lead to increased risk of infection. Additionally, wound healing is associated with less acidic pH which can influence microbial colonisation and infection (Rippke et al., 2018). In the urinary tract the pH of normal urine is slightly acidic (pH 6–7.5), but a range as wide as 4.0–8.0 is normal. During infection this can rise to 9.0 (e.g., “urea-splitting” pathogens such as *Proteus* spp., *Klebsiella pneumoniae*, or *Ureaplasma urealyticum*) and is a clear indicator of a UTI (Bono and Reygaert, 2019). Conventional AST is conducted at pH 7.2–7.4 for bacteria and pH 7 for fungi which may be appropriate for many infections, but consideration of the pH (and the buffer used to attain this) for testing should be taken into account when investigating target pathogens or infections at sites with different pH.

### Oxygen (Hyper-, Norm-, and Hypoxia)

Antimicrobial susceptibility testing is normally conducted under conditions of normoxia, yet in tissues the amount of oxygen range between <1 and 11% oxygen, whereas *in vitro* experiments are normally performed in 19.95% oxygen, an artificially high concentration relative to tissue concentrations. For example, in normal air the oxygen partial pressure is 160 mmHg, whereas in alveoli, this is reduced to 110 mmHg, in the brain 23–48 mmHg and in the colon, only 3–4 mmHg (Carreau et al., 2011). Areas of hypoxia are features of sites of bacterial infection, healing wounds and other diseased tissues (Murdoch et al., 2005) and hypoxia can induce the expression of hBD-2 (Nickel et al., 2012). Thus, physiological oxygen concentrations vary widely, yet are largely not considered in the context of antimicrobial efficacy, unless specifically considering activity against anaerobes or microaerophiles. Given the membrane disruption mechanism of action of many AMP, should oxygen be a factor affecting their activity? The plectasin-derived AMP, NZ2114 and MP1102, were bactericidal by membrane lysis vs. *Clostridium perfringens* under anaerobic conditions (>3 log kill in <60 min) (Zheng et al., 2017), whereas LL-37 and hBD-3 (<5 mg/L) were bactericidal against *C. difficile* under anaerobic conditions (Nuding et al., 2014). The activity of human defensins against anaerobic bacteria revealed that human α-defensin 5 and hBD-1 were minimally active against a panel of 25 strict anaerobes, hBD-2 demonstrated relatively weak activity against most strict anaerobes, whereas hBD-3 was active against 18 of 25 strict anaerobes tested (Nuding et al., 2009). Interestingly, human α-defensin 6 (HD-6), the second most abundant AMP produced by Paneth cells in the small intestine (Wehkamp et al., 2005), does not demonstrate direct antimicrobial activity under standard aerobic conditions, but demonstrated direct killing of *Bifidobacterium adolescentis, Lactobacillus acidophilus*, and *Bif. breve, Bif. longum* and *Streptococcus salivarius* subsp. *thermophilus* under reducing conditions (mimicking anaerobiosis) (Schroeder et al., 2015). Oh and co-workers tested 16 CAMEL peptides (cecropin-melittin hybrids) against a selection of anaerobes (*Peptostreptococcus* spp., *C. difficile, Bacteroides fragilis, Prevotella*, spp., *Fusobacterium nucleatum* and *Propionibacterium* spp.; *n* = 60) under anaerobic conditions and all were active (MIC_90_ = 1–32 mg/L) (Oh et al., 2000). Piscidins and ixosin are AMP that contain the copper- and nickel-binding ATCUN motif. Bactericidal activity under aerobic conditions is enhanced when these AMP bind copper, but under anaerobic conditions two piscidins (p1 and p3) and ixosin retain activity, but this does not depend on the presence of copper ions (Libardo et al., 2016; Oludiran et al., 2019). Thus, it would appear that antimicrobial activity of AMP under anaerobic conditions is dependent on the AMP used and for the purposes of AMP vs. anaerobes would need to be assessed on a case-by-case basis.

### Proteolysis

Susceptibility to proteolysis is often viewed as one of the most significant limitations when trying to develop peptide drugs, including AMP (Vlieghe et al., 2010; Lecaille et al., 2016; Starr and Wimley, 2017). When conducting AST with AMP, protease production by the pathogen of interest is an obvious potential cause of reduction in/or loss of activity (Stumpe et al., 1998; Schmidtchen et al., 2002; Nesuta et al., 2017; Rapala-Kozik et al., 2018). Additionally, if testing were to be carried out in biological matrices other than growth media (e.g., blood, saliva etc.), then proteolysis by relevant host proteases could adversely affect AMP activity (Knappe et al., 2010; Lecaille et al., 2016; Starr et al., 2016; Bottger et al., 2017; Starr and Wimley, 2017). To determine whether proteolysis could occur during AST, protease inhibitors could be included in the system (Shin et al., 2010). However, this is not necessarily as simple as it appears. A number of protease inhibitors, including EDTA and citrate, can enhance AMP activity by chelation of metal ions, or by other unknown mechanisms, and this activity would need to be determined prior to their use in AST (Wei and Bobek, 2005; Walkenhorst et al., 2014; Maisetta et al., 2017; Umerska et al., 2018; Grassi et al., 2019).

### Bicarbonate

Bicarbonate (NaHCO_3_) is relatively common in mammalian tissues (NaHCO_3_; 24.90 ± 1.79 mM in human blood Wishart et al., 2018) and the bicarbonate buffer system, sodium bicarbonate in balance with carbonic acid, helps to maintain the physiological pH, including blood, interstitial fluid and the upper gastro-intestinal tract (Boron and Boulpaep, 2005). However, bicarbonate warrants additional consideration beyond its capacity for maintaining pH homeostasis. Despite its importance physiologically, it is not routinely considered when conducting AST or in the maintenance of pH during AST. Even though AST of fungi uses RPMI-1640 liquid medium, a mammalian cell culture medium, this is buffered to pH 7.0 with MOPS (CLSI, 2017a,c), rather than sodium bicarbonate as it would be when culturing mammalian cells and using a CO_2_ incubator (5% CO_2_). Sodium bicarbonate has antibacterial, antifungal and antibiofilm properties of its own, but only at supra-physiological concentrations (≥50 mM) (Corral et al., 1988; Xie et al., 2010; Letscher-Bru et al., 2013; Dobay et al., 2018; Farha et al., 2018). Bicarbonate acts as a selective dissipater of the trans-membrane pH gradient, a component of the proton motive force (along with the membrane potential) and can enhance the activity of AMP, including LL-37, α-defensin, indolicidin, protegrin and bactenecin and selected antibiotics, including aminoglycosides, macrolides and selected fluoroquinolones. The activity of AMP was enhanced as both the AMP and bicarbonate perturb bacterial membrane potential and in the case of antibiotics, enhancement of activity was predominantly limited to those whose uptake is driven by the membrane potential (Farha et al., 2018). Interestingly, tobramycin (aminoglycoside) activity against isolates of *P. aeruginosa* was enhanced in the presence of bicarbonate against planktonic cells, but the combination promoted biofilm growth (Kaushik et al., 2016). The relevance of bicarbonate addition during AST of selected antibiotics against bacteria has been investigated. When CA-MH broth was supplemented with physiological levels of bicarbonate, this improved the predictive value of AST for treatment of *in vivo* infections for a number of antibiotic and pathogen combinations, potentially due to structural changes to bacteria and changes in gene expression (Ersoy et al., 2017). When analysing the effect of bicarbonate on the sensitivity of isolates of MRSA to anti-staphylococcal β-lactams, two phenotypes became apparent; those that became susceptible on bicarbonate supplementation and those that were unaffected. In the isolates that became susceptible, bicarbonate supplementation caused reduced expression of *mecA* and *sarA*, which led to decreased production of penicillin-binding protein 2a and correlated with sensitivity to β-lactams in a rabbit infective endocarditis model comparable to that of MSSA isolates. Additionally, bicarbonate responsive isolates demonstrated lower survival when the β-lactam was combined with LL-37 *in vitro* and this may have enhanced the efficacy seen *in vivo* (Ersoy et al., 2019).

Dorschner and colleagues observed inhibition of *S. aureus* growth by LL-37 was greater in MEM, a cell culture medium, when compared with Tryptone Soy Broth (a nutrient-rich bacterial growth medium) containing the same concentrations of NaCl and FBS (Dorschner et al., 2006). By analysis of individual components of MEM, they determined that it was the presence of physiological concentrations of sodium bicarbonate that enhanced membrane-permeabilising activity of the following AMP: LL-37, mCRAMP (murine cathelicidin-related antimicrobial peptide), PR-39 (a porcine cathelicidin), hBD-2 (human β-defensin 2), but not dermcidin (an anionic AMP from human skin and sweat) and not in a pH-dependent manner. The presence of bicarbonate; may also have ameliorated the inhibitory effect of the 150 mM NaCl in the medium used, as NaCl concentrations of >50 mM can inhibit the activity of many AMP (Goldman et al., 1997; Travis et al., 2000). Culturing *E. coli* O29 in the presence of NaHCO_3_ affected the expression of a number of virulence-related genes that could increase susceptibility to AMP (Dorschner et al., 2006). The addition of 25 mM bicarbonate enhanced the activity of the AMP tritrpticin against the protozoan *Trichomonas vaginalis* (Infante et al., 2011). Conversely, selected *S. aureus* small-colony variants (SCV) were less susceptible to LL-37 when incubated in the presence of 50 mM NaHCO_3_; (~2 x physiological concentration) (Zhang et al., 2018), although the effect of physiological bicarbonate concentrations was not examined. Small colony variants (SCV) are slow-growing sub-populations of bacteria with altered metabolism and reduced antibiotic susceptibility which, in the case of *S. aureus*, can cause persisting and recurrent infections (Baumert et al., 2002). *S. aureus* SCV are already known to be less susceptible to a number of AMP when compared to wild-type cells (Koo et al., 1996; Sadowska et al., 2002; Samuelsen et al., 2005), although this effect was not observed when a cationic antimicrobial polypeptide was tested against *S. aureus* SCV (Mercer et al., 2017). It would therefore be relevant to examine the effects of media supplementation with physiological concentrations of bicarbonate (25 mM; 2.1 g/L) when conducting AST with AMP to generate results possibly more predictive of efficacy *in vivo*. Such results must be viewed with caution, however, in light of the biofilm promoting effects in combination with tobramycin (Kaushik et al., 2016).

### Temperature

The effect of temperature on the activity of AMP during AST has not been widely investigated, as most AMP are intended for use against infectious diseases AST is predominantly conducted at body temperature (~37°C) or at temperatures recommended in CLSI or EUCAST guidelines (30–37°C). Thus, it seems most relevant to conduct AST at physiological temperatures. At low temperatures membrane bilayers undergo a reversible change of state from a fluid (disordered, liquid crystalline) to a non-fluid (ordered, gel) array of the fatty acyl chains (de Mendoza, 2014) and this increase in membrane rigidity can lead to reduced AMP efficacy/resistance (Cole and Nizet, 2016; Joo et al., 2016). Interestingly, the *P. aeruginosa* quorum-sensing molecule 2-n-heptyl-4-hydroxyquinoline N-oxide (HQNO) increases membrane fluidity in *S. aureus*, so it would be intriguing to determine whether this increases the sensitivity of *S. aureus* to AMP (Orazi et al., 2019). HQNO can also induce *S. aureus* to adopt the SCV phenotype (Hoffman et al., 2006) and this may also affect AMP activity. The effect of high and low temperatures on storage or preparation of AMP, as well as activity, has received consideration in a number of studies (Wei et al., 2007; Zhang et al., 2011; Ji et al., 2014; Lee et al., 2014; Jiao et al., 2019). For example, many AMP demonstrate stability at temperatures below 100°C. For example, the thermal stability of 8 AMP (Cap18, Cap11, Cap-11-1-18m2, Cecropin B, Cecropin P1, Indolicidin and Sub5) was assessed by heating them to 70 or 90°C for up to 30 min before conducting AST. All AMP were stable following heating, with only Sub5 demonstrating an MIC increase (4 to 8 mg/L) after being heated to 70°C for 30 min, but not when heated to 90°C (Ebbensgaard et al., 2015). However, this is not the case for all AMP. The AMP epinecidin-1 was not stable at elevated temperatures and demonstrated a 32 - >64-fold increase in MIC against *S. aureus* following incubation at 60–100°C for 5 min (Huang et al., 2017). As is well-documented, AMP have been isolated from almost all life-forms, including arctic and Antarctic fish. Moronecidin (isolated from the hybrid striped bass) and 2 derivatives were assessed for efficacy against *Psychrobacter* spp. PAMC25501 at 5–15°C and *E. coli* DH5α at 15–37°C and no differences in MIC were observed at different temperatures (Shin et al., 2017). The activity of the piscidin-like AMP, chionodracine (isolated from the icefish *Chiondraco hamatus*) was more active at 25 than 37°C against *E. coli* (MIC 20 and 5 mg/L, respectively) and *B. cereus* (MIC 10 and 5 mg/L, respectively) (Buonocore et al., 2012), although this may reflect adaptation to the low temperature environment from which they were isolated.

### Metal Ions

Transition metal ions influence the activity of AMP in a variety of ways. In some cases, the activity of AMP are dependent on the presence of metal ions (Paulmann et al., 2012; Melino et al., 2014; Alexander et al., 2018; Jezowska-Bojczuk and Stokowa-Soltys, 2018; Agbale et al., 2019), whereas in others the presence of metal ions can cause reduction, or even complete abrogation, of activity (Friedrich et al., 1999; Deslouches et al., 2005). In most circumstances, the effect of metal ions must be considered on a case-by-case basis as different ions will produce a distinct effect. For example, LL-37 is inactive in the presence of ≥3 μM MgCl_2_, whereas the *de novo* designed AMP, WLBU2, remains active at the same concentration. LL-37 is also less potent in the presence of ≥1 μM CaCl_2_, whereas the MIC of WLBU2 increases by ~4-fold in the presence of ≥6 μM CaCl_2_, (Deslouches et al., 2005). Activity of the cecropin-melittin hybrid peptide variants was ≥4-fold increased in the presence of 3–5 mM MgCl_2_ (representing the serum concentration of divalent cations), although one variant is insensitive to the effects of 3 mM MgCl_2_, but not to 5 mM MgCl_2_ (Friedrich et al., 1999).

Susceptibility testing of *K. pneumoniae* against tetracycline in tissue culture medium predicts resistance, whereas testing in MHB and LPM pH 5.5 media predicts susceptibility. Mice infected with *K. pneumoniae* and treated with tetracycline survive infection mirroring the results obtained in pH 5.5 media, conditions that resemble best the environment in which tetracycline interacts with this pathogen since *K. pneumoniae* resides within macrophage phagosomes (Ersoy et al., 2017). This example highlights the importance of mimicking the environment of phagosomes when dealing with intracellular pathogens. Besides *K. pneumoniae*, other important pathogens that thrive in an intracellular environment include *Salmonella* spp. (de Jong et al., 2012; Helaine et al., 2014), *Mycobacterium tuberculosis* (Pieters, 2008) and *Legionella pneumophila* (Escoll et al., 2013). *Streptococcus pyogenes* is also suspected of sharing this lifestyle (Hertzen et al., 2012).

After phagosomes internalize their cargo, acidification of the interior takes place in a rapid process. For example, the phagocytic compartment of a bone marrow-derived macrophage phagosome following internalization of an immunoglobulin G-coated particle reaches a pH of 5.0 or below within 10–12 min of internalization of an immunoglobulin G (IgG)-coated particle (Yates et al., 2005). The acidic, hydrolytically competent environment of the phagolysosome in combination with other antimicrobial effectors typically lead to the death and digestion of most non-pathogenic microbes. It is in this environment that the intracellular pathogens mentioned above survive and thrive, and more importantly, it is where the interaction between an antimicrobial agent and the pathogen will take place. Besides the low pH, other antimicrobial effectors fill the phagocytic compartment, including metal ions.

Metal ions such as copper and zinc have been observed at high concentrations in the phagocytic milieu as a response to certain types of infections. Wagner et al. using a hard x-ray microprobe with suboptical resolution reported that upon infection by the human pathogens *M. tuberculosis* and *M. avium* or with avirulent *M. smegmatis* the concentration of copper and zinc ions within phagolysosomes of peritoneal macrophages can be as high as 426 ± 393 μM and 459 ± 271 μM, respectively (Table 3) (Wagner et al., 2005). Additional indirect evidence for the presence of copper ions in the intracellular battlefield against pathogens include the observation that in IFN-γ and LPS-activated macrophages the levels of the Ctr1 Cu importer are elevated (White et al., 2009). Moreover, the Cu pump ATP7A is overexpressed and localized to the phagolysosome, suggesting accumulation of Cu within this compartment. Interestingly, macrophage exposure to the Cu chelator tetrathiomolybdate (TTM) results in increased survival of *S. typhimurium* (Achard et al., 2012). Besides the quantitative determination of Zn by Wagner and co-workers, there is additional evidence for a role of host Zn in the direct overload killing of invading pathogens (Djoko et al., 2015). Using a Zn-responsive fluorescent probe, it was observed that infection of human macrophages with either *E. coli* or *M. tuberculosis* leads to an increase in the intracellular levels of Zn (Botella et al., 2011). Consistent with the hypothesis that Zn directly kills phagocytosed pathogens, it was observed that bacterial mutants defective in Zn export (zntA and ctpC in *E. coli* and *M. tuberculosis*, respectively) showed decrease survival within these human macrophages. Similar observations of increased Zn concentrations within phagocytic cells have been observed upon infection with *Histoplasma capsulatum* (Subramanian Vignesh et al., 2013), and *Streptococcus pyogenes* (Ong et al., 2015). As observed for *E. coli* and *M. tuberculosis*, S. *pyogenes* mutants defective in Zn efflux had a lower survival within the hosts.

**Table 3:** Concentration of copper and zinc ions within phagolysosomes of peritoneal macrophages during infection by three *Mycobacterium* spp.

**Element**	**Time**	***M. smegmatis***	***M. avium***	***M. tuberculosis***
Cu	1 h	9.9 ± 5.5 μM	28.3 ± 11.4 μM	426 ± 393 μM
	24 h	24.8 ± 0.65 μM	17.3 ± 10.3 μM	24.7 ± 9.5 μM
Zn	1 h	70.5 ± 37.3 μM	134.6 ± 38.8 μM	37.8 ± 25.2 μM
	24 h	260 ± 117 μM	120.8 ± 31.1 μM	459 ± 271 μM

*Concentrations were determined using hard x-ray microprobe with sub-optical resolution (Wagner et al., 2005)*.

There is also evidence for removal of Zn ions from phagosomes during infections and excellent reviews on the topic exist. In this work, we wanted to limit ourselves to those environments in which the concentration of Zn ions increased.

Besides the phagocytic environment, there are other sites of microbial infection in which copper and zinc ions are found at concentrations in which they can affect the activity of antimicrobial agents. For instance, during urinary tract infection by the pathogens *Proteus mirabilis* and *K. pneumoniae*, copper is found at micromolar concentration as a host response to the infection (Hyre et al., 2017). Additional *in vivo* studies demonstrated that Cu-deficient mice are more susceptible to uropathogenic *E. coli* infection, indicating that copper release into urine is an important innate defence mechanism. An additional human fluid that contains metal ions at concentrations high enough to affect the antimicrobial activity of antibiotics is sweat (Troy et al., 2007). Copper concentrations range from 4.6 ± 0.4 μM to 20 ± 10 μM, whereas zinc concentrations can be as high as 630 μM. Copper levels in human saliva also have been reported to range from 1.6 μM to 7.5 μM (Dreizen et al., 1952; Borello, 1976). Human saliva also contains zinc ions with reports indicating a maximum concentration of 6.7 mM (Sejdini et al., 2018). Clearly, copper and zinc ion interactions with antimicrobial agents is plausible outside phagocytic compartments.

Antibiotics, with their richness of functional groups, are poised for metal ion coordination. The result of this interaction can range from antagonism to synergism, although the former is the most common outcome. Back in 1946, Eisner et al. reported the inactivation of penicillin by zinc salts (sulphate, acetate, chloride, and oxide) (Eisner and Porzecanski, 1946). Amoxicillin and ampicillin are also readily degraded by zinc ions (Navarro et al., 2003). Tetracycline and several of its derivatives avidly bind copper and zinc ions to form 2:1 complexes (Doluisio and Martin, 1963; Brion et al., 1985). Indeed, the formation of tetracycline-zinc complexes is suspected to affect the metabolism of the drug and adversely impact its antibiotic activity (Doluisio and Martin, 1963; Brion et al., 1985). Other antibiotics are known to bind metal ions (e.g., quinolones Seedher and Agarwal, 2010; Uivarosi, 2013 and aminoglycosides Lesniak et al., 2003). Interestingly, many chelates of quinolones show equal or enhanced activity compared to that of the parent antibiotic. The reason for the superior activity of the quinolone-metal complexes is not clear. Overall, the impact of metal binding on the activity of many antibiotics is undeniable and deserves attention during susceptibility assays.

**Figure 2:**
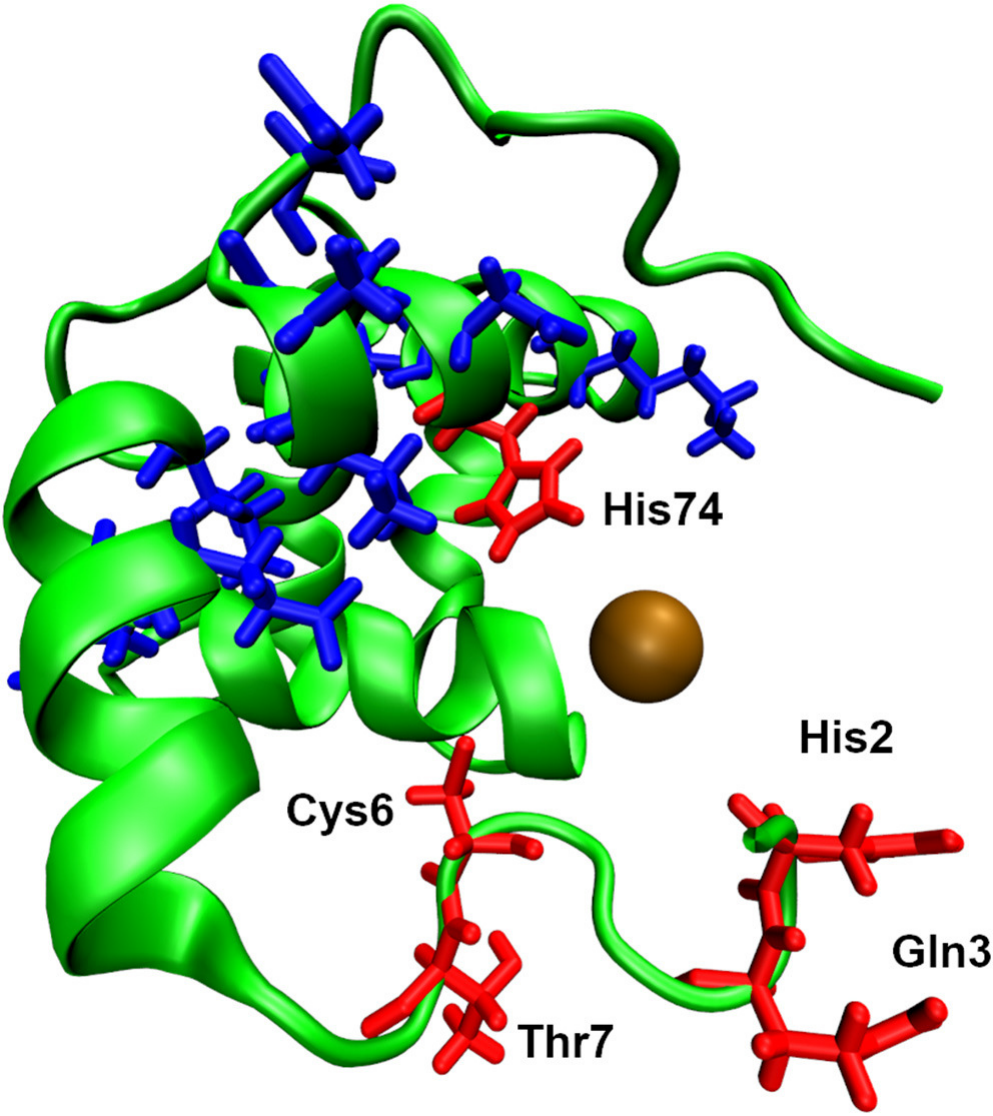
Structure of microplusin solved by NMR solution spectroscopy (PDB ID: 2KNJ). For illustration purposes the Cu^2+^ ion has been added, shown as brown sphere, to its putative binding site. The four labelled residues, shown as red liquorice, are highly likely to bind Cu^2+^ ion due to their proximity to the ion. The other residues, shown as blue liquorice, are also capable of binding Cu ion, as suggested by the NMR experiment (Silva et al., 2009).

The effect on the antimicrobial activity of AMP of the presence of Cu and Zn ions in the assay media is difficult to predict. Typically, compounds that act as simple metal chelators will see a decrease in their antimicrobial activity as the concentration of chelated ions increase. An example of an antimicrobial peptide that acts as a metal chelator is the tick peptide microplusin, which specifically chelates Cu^2+^ ions (Figure 2). The opposite effect, that is the increase in activity in the presence of these metal ions, also takes place although it has received less attention. In the case of copper ions, several antimicrobial peptides have been recently reported to utilize this ion to enhance its bactericidal activity (Libardo et al., 2014, 2015, 2016, 2017; Hayden et al., 2015; Alexander et al., 2017). One of the earliest examples of the AMP requirement for Zn^2+^ is that of bacitracin, a mixture of chemically related dodecapeptides isolated from *Bacillus subtilis* and *Bacillus licheniformis*. Bacitracin, a drug and food additive, has a multimodal mechanism of action (Storm and Strominger, 1973; Storm, 1974; Karala and Ruddock, 2010; Dickerhof et al., 2011). There is consensus in that the main mode of action involves the inhibition of bacterial cell-wall biosynthesis by targeting extracellular, membrane-associated pyrophosphate groups. Remarkably, recognition of the pyrophosphate group of the lipid target is mediated by a zinc ion which organizes the N-terminal region of the peptide and neutralizes the large charge of the ligand, thus allowing direct antibiotic–lipid interactions (Storm and Strominger, 1973; Economou et al., 2013). Other peptides with enhanced its antimicrobial activity in the presence of Zn^2+^are the anionic human AMP DCD-1 and its derivative DCD-1L. Both peptides are present in eccrine sweat and are highly active at pH values that range from 5.5 to 7.4 and retain their activities against several microorganisms when the ionic strength of the medium is increased by adding up to 150 mM NaCl (Dennison et al., 2006; Song et al., 2013; Becucci et al., 2014; Libardo and Angeles-Boza, 2014). The Zn^2+^ ions are required for the formation of ion channels as they promote the formation of a trimer of dimers. One final example that is worth mentioning corresponds to the synthetic ^*^ARVA peptides, which contain no histidines, that were selected from a combinatorial library and shown to be synergistic with copper and zinc ions (Walkenhorst et al., 2014; Walkenhorst, 2016). Thus, there is mounting evidence that the activity of AMP is affected by the presence of copper and zinc ions.

An important question that must be asked at this point is whether the presence of other divalent cations would resemble the effect of Cu and Zn ions? The short answer is no, as the effects on the activity of AMP by Ca^2+^ and Mg^2+^ ions often differ from those effects that originate from the presence of Cu and Zn ions. For example, the presence of CaCl_2_ and MgCl_2_ does not affect the activity of microplusin; however, similar concentrations of CuCl_2_ have a negative impact in the activity of the AMP (Silva et al., 2009). Similarly, the presence of Ca^2+^ ions decrease the antimicrobial activity of clavanin A, an AMP found in phagocytic cells, whereas Zn^2+^ ions increased its activity (Juliano et al., 2017). In short, Ca^2+^ and Mg^2+^ ions do not have the same effect as Cu and Zn ions in the antimicrobial activity of AMP.

Excluding the highest and lowest values of the metal concentrations in the set of values shown in Table 3, the average concentrations of Cu and Zn ions are 24 and 146 μM, respectively; therefore, we recommend using Cu and Zn concentrations of 25 and 150 μM, respectively if the phagosomal environment is to be mimicked. [Zn^2+^] = 150 μM is well above the concentration required to maximize the potency of clavanin A 16-fold at pH 5.5 (Juliano et al., 2017). Likewise, 150 μM is in the upper range of the Zn concentrations that were synergistic with ^*^ARVA peptides (Walkenhorst et al., 2014). Since Ersoy has successfully used LPM (low magnesium medium) media at pH 5.5 to mimic the phagolysosome compartment, it is suggested that using this media in combination with Cu and Zn ions. CuCl_2_ and ZnCl_2_ can be used as sources for these ions. If after performing the assay in the presence of copper and zinc ions as suggested above one is suspicious of synergy between any of these metal ions and the AMP, the best assay to follow is the one described by Walkenhorst et al. which allows the determination of FIC values using a 96-well plate. CuCl_2_ and ZnCl_2_ were used in liquid test medium (LTM) and the procedure is clearly described in the paper of Walkenhorst and co-workers (Walkenhorst et al., 2014).

### Salt (NaCl)

The effect of NaCl on AMP activity needs to be taken into account depending on the site of infection as the concentration can vary significantly, from ~140 mM in blood (Li et al., 2016), 12–52 mM in saliva (Ferguson and Botchway, 1979) and 8–14 mmol/mmol creatinine in urine (Sasaki et al., 1998), whereas in certain infectious disease states this can be much higher (up to 180 mM in CF sputum Grandjean Lapierre et al., 2017 and 300 mM in infected urine Withman et al., 2013). Distinct from other metal ions and salts, some AMP are sensitive to higher salt concentrations. For example, human β-defensin 1 retains activity at low NaCl concentrations (≤50 mM), but this is rapidly lost when the NaCl concentration increases above 75 mM (Goldman et al., 1997), which are physiologically relevant (Wishart et al., 2018). This sensitivity was to the Na^+^ ion, not the Cl^−^ ion. Similarly, the activity of LL-37 is reduced significantly in the presence of 100 mM NaCl (Turner et al., 1998) and the activities of defensins are similarly reduced (Bals et al., 1998; Garcia et al., 2001). The tryptophan-rich AMP, Pac-525, demonstrated reduced activity in the presence of 100–300 mM NaCl, whereas the Pac-525 derivative, d-Nal-Pac-525 (all d-amino acids and tryptophan residues replaced with d-β-naphthylalanine) was not sensitive to these NaCl concentrations (Wang et al., 2009). The histatin derivative P-113 was also sensitive to high NaCl concentrations (as well as additional Ca^2+^ and Mg^2+^ in the media) (Rothstein et al., 2001). Conversely, clavanin retained activity in 100 mM NaCl and Clavanin AK retained activity in 300 mM NaCl (Lee et al., 1997), the *de novo* designed peptide SHAP1 retained activity in the presence of 200 mM NaCl (Kim et al., 2014), the *Streptococcus mutans*-specific AMP IMB-2 retained ~85% activity in the presence of 134 mM NaCl (Mai et al., 2011) and the RR12 AMP retained activity in 300 mM NaCl (Mohanram and Bhattacharjya, 2016). Therefore, when considering AST of AMP, the concentration of NaCl at the site of infection is an important factor and the effects of NaCl on AMP activity will need to be assessed on a case-by-case basis. If the tested AMP is salt-sensitive, there are a number of possible strategies available to reduce salt-sensitivity (Harwig et al., 1996; Friedrich et al., 1999; Tam et al., 2000; Park et al., 2004; Yu et al., 2011; Chu et al., 2013).

### Inoculum Size

The efficacy of an antimicrobial during AST will necessarily depend on the size of inoculum tested. The inoculum effect is a well-documented laboratory phenomenon that can be described as a significant elevation in antibiotic MIC when the number of inoculated microorganisms is increased (Brook, 1989; Smith and Kirby, 2018; Idelevich and Becker, 2019). Elevated MIC (reduced efficacy) values as a function of increased cell density has been observed for a limited number of AMP, including LL-37 (Snoussi et al., 2018), ARVA (Starr et al., 2016) and DNS-PMAP 23 (Savini et al., 2017). In cultures with a low inoculum density, MICs of AMP and antibiotics can reach a plateau value that is independent of cell density (Udekwu et al., 2009; Savini et al., 2017).

An additional consideration must be the presence of host cells as these can effectively contribute to the inoculum effect. For example, conducting AST in the presence of host cells, e.g., red blood cells, can cause a loss of activity against the target pathogen due to binding of the AMP to these host cells, even if selectivity is lower than for the pathogen (Starr et al., 2016). However, bactericidal concentrations of AMP can be achieved in the presence of host cells (Savini et al., 2017) and this should also be possible clinically. Thus, when conducting AST of AMP the size microbial inoculum (cfu/ml) must be given due consideration and a balance reached between low cell density resulting in efficacy that is unachievable *in vivo* and high cell density that results in under-estimation of activity and the potential loss of promising therapeutic candidates. As a starting point the cell densities specified in CLSI, ISO and EUCAST documents (Table 1) are not unreasonable starting points, but consideration should also be given to the pathogen and site of infection, as *in vivo* information may be available to better guide the likely cell density during infection and this would clearly be more relevant to take into consideration.

### Growth Phase: Biofilms, Persister Cells Spores and Small Colony Variants (SCV)

When conducting broth dilution AST using CLSI or EUCAST protocols, bacteria are normally prepared when actively growing (exponential phase) or in the stationary phase, depending on whether the inoculum is prepared by the broth culture or colony suspension methods, respectively (CLSI, 2018a; ISO, 2019), but the potential influence of growth phase on AST results is not taken into consideration. Similarly, the growth phase is not factored in when preparing yeast inocula (CLSI, 2017a). Conversely, AST of filamentous fungi starts with an inoculum prepared from a spore suspension (CLSI, 2017c) that can take a number of hours to germinate into actively growing hyphae. In most cases, AST of conventional antibiotics and antifungals requires actively metabolising/growing cells, as their mechanism of action relies on physiological processes, including DNA/RNA replication (fluoroquinolones and flucytosine), protein biosynthesis (tetracyclines and macrolides), cell wall biosynthesis (β-lactams and echinocandins) and membrane biosynthesis (polyenes and azoles) (Silver, 2011; Perfect, 2017). Therefore, it is important that microorganisms are actively growing, or have the capacity to do so, when AST is performed. Membrane-active AMP, on the other hand, are active against metabolically inactive as well as active cells; a major potential therapeutic advantage, e.g., (Mercer et al., 2017). As such, it is not critical that cells be metabolically active when AST is conducted for these AMP, and it is only by following convention than most AST of AMP is done on actively metabolising cells. At sites of infection, microorganisms are rarely found growing in the exponential phase on nutrient-rich media sources; therefore, the importance of microbes existing in other growth phases requires consideration and there is evidence that slower growing pathogens are more virulent than faster growing pathogens (Leggett et al., 2017). Additionally, a number of different parameters influencing growth phase can alter virulence gene expression in pathogens, including toxin and adhesin production in *E. coli* (Crofts et al., 2018) and hyphal development in *Candida* spp. (Su et al., 2018).

Given the heterogeneity of microbial populations within an infection site, a range of cell morphologies and environmental conditions will exist and these will have an impact on antimicrobial susceptibility. Given this heterogeneity, it is relevant to test antimicrobial efficacy, including that of AMP, vs. microbes existing in different growth states (Radlinski and Conlon, 2018; Dewachter et al., 2019). Specialised slow or non-growing forms of microbial pathogens include biofilms (Desai et al., 2014; Reichhardt et al., 2016; Koo et al., 2017; Wolfmeier et al., 2018; Orazi and O'Toole, 2019), spores (Setlow, 2014; Gil et al., 2017), persister cells (Fisher et al., 2017; Wuyts et al., 2018; Balaban et al., 2019) and small colony variants (SCV) (Proctor et al., 2006; Johns et al., 2015). In some cases, there are even combinations of these cells, e.g., persister cells and/or SCV within biofilms (Mirani et al., 2015; Waters et al., 2016; Wuyts et al., 2018; Yan and Bassler, 2019). A common feature of microbial growth in these forms is normally reduced susceptibility to antibiotics and antifungals. The efficacy of AMP has been tested against cells demonstrating differing growth modes.

At the simplest level, a microbial biofilm is a surface-associated community of microorganisms surrounded by an extracellular polymeric matrix. It is estimated that more than 80% of microbial infections are caused by microbes growing as biofilms (Romling and Balsalobre, 2012), and therefore their determination of their susceptibility to antimicrobial agents, including AMP, is of paramount importance when developing novel antimicrobial agents. An increasing understanding of biofilm infections has led to the appreciation that many infections are polymicrobial in nature and may contain diverse species of bacteria, fungi and viruses (Peters et al., 2012; Wolcott et al., 2013; Mihai et al., 2015; Todd and Peters, 2019) and a number of models of polymicrobial biofilms have recently been developed (Gabrilska and Rumbaugh, 2015). Polymicrobial biofilms are of relevance in many infections, including chronic wound infections (Clinton and Carter, 2015), CF lung infections (Lopes et al., 2015), bacterial vaginosis (Jung et al., 2017) and medical device-associated infections (Wi and Patel, 2018). Microbes growing in biofilms can be up to 1000-fold more tolerant to antimicrobials than their planktonically growing counterparts (Costerton et al., 1995; Hoiby et al., 2010), so more effective therapeutic options are urgently required. A number of mechanisms are responsible for biofilm antibiotic-tolerance including; (1) reduced diffusion of antibiotics through the biofilm matrix, (2) sequestration of antibiotics by the biofilm matrix; (3) presence of slow-growing and persister cells (see below) refractory to antibiotics targeting bacterial metabolism; and (4) increased exchange of antibiotic resistance genes on mobile genetic elements by cells in close proximity (Stewart and Costerton, 2001; Hall and Mah, 2017; Orazi and O'Toole, 2019). The potential role of AMP as therapeutics to treat biofilm infections has received significantly more attention than activity vs. spores, persister cells or SCV and has been the subject of several review articles in the last few years (Batoni et al., 2016; de la Fuente-Nunez et al., 2016; Pletzer and Hancock, 2016; Delattin et al., 2017; Grassi et al., 2017b; van Dijck et al., 2018; Von Borowski et al., 2018; Yasir et al., 2018; Shahrour et al., 2019). A detailed analysis of the potential role of AMP in the treatment of biofilm infections is beyond the scope of this manuscript, but some key points with respect to AST will be addressed in the following paragraphs. Membrane disruption is the most common mechanism of action of cationic AMP and remains important in some instances for anti-biofilm properties of AMP, but when assessing anti-biofilm properties, other attributes of AMP may be of equal or greater importance (Yasir et al., 2018), including; (1) blocking of/interference with bacterial cell signalling systems, e.g., LL-37 (Overhage et al., 2008); (2) degradation of the biofilm matrix, e.g., piscidin-3 (Libardo et al., 2017); (3) inhibition of the alarmone system to avoid the bacterial stringent response, e.g., peptide 1018 (de la Fuente-Nunez et al., 2014); (4) downregulation of genes responsible for biofilm formation, e.g., hBD-3 (Zhu et al., 2013); and (5) immunomodulatory properties may also confer additional *in vivo* anti-biofilm properties. Interestingly, some AMP are able to prevent biofilm formation at concentrations significantly below their MIC, or have anti-biofilm properties, but without antibacterial activity (for examples see (Pletzer and Hancock, 2016; Mercer et al., 2017; Haney et al., 2018a; Shahrour et al., 2019), indicating a separate mechanism of action from membrane disruption/direct killing. The membrane disruption properties of AMP have been the main focus of this manuscript and will remain so for the discussion of AST of AMP and biofilms.

Examples of AMP that can kill biofilm microbes by membrane permeabilization include the eosinophil cationic protein-derived AMP, RN3(5-17P22-36), that demonstrated membrane-permeabilising activity (Sytox green membrane permeabilization) against established biofilms of *P. aeruginosa*, albeit at 2–8-fold higher concentrations than required for the same activity vs. planktonic *P. aeruginosa* (Pulido et al., 2016). The esculentin-1a-derived AMP, Esc(1-21), also caused permeabilization of the membranes of planktonic cells and biofilms of *P. aeruginosa* PAO1 (Sytox green membrane permeabilization and β-galactosidase release) (Luca et al., 2013), and LL-37 and selected truncated versions (LL-31, LL7-37, LL13-37, and LL7-31), albeit at relatively high concentrations (20–100 μM) (measured by propidium iodide (PI) uptake) against pre-grown *P. aeruginosa* PAO1 biofilms (Nagant et al., 2012). The lactoferricin-derived AMP, LF11-215, LF11-324 and a lipopeptide derivative, DI-MB-LF11-324, caused membrane permeabilization of *P. aeruginosa* PAO1 in biofilms (PI uptake) at 10 × MIC concentrations (Sanchez-Gomez et al., 2015). The related AMP Seg6L and Seg6D both demonstrated antibiofilm properties with Seg6D predominantly causing cell lysis, whereas Seg6L degraded the biofilm by detaching live cells, rather than direct killing, demonstrating that relatively minor changes to AMP composition (substitution of 5 Seg6L amino acids for D-isoform amino acids) can substantially affect antimicrobial properties (Segev-Zarko et al., 2015). Some AMP are also membrane-active against fungal biofilms (van Dijck et al., 2018). Tyrocidines are cationic cyclodecapeptides with broad-spectrum antimicrobial activity and the tyrocidines (TrcA, TrcB, and TrcC) were able to disrupt the membrane of *Candida albicans* growing as biofilms (PI uptake) at concentrations similar to the planktonic MIC (~12.5 μM), but significantly higher concentrations were required to eradicate biofilms (Troskie et al., 2014). Similarly, the peptidomimetic mPE was able to disrupt the membrane of *Candida albicans* growing as biofilms (PI uptake) at concentrations 6–12-fold higher (50–100 mg/L) than the MIC vs. planktonic cells (Hua et al., 2010). Studies of the activity of AMP vs. polymicrobial biofilms are less frequently reported, but the Herpes simplex-derived AMP gH625-GCGKKKK was able to prevent formation of mixed species biofilms consisting of *Candida tropicalis* and *S. aureus* or *C. tropicalis* and *Serratia marcescens* as well as eradicating mixed biofilms containing these microbes (de Alteriis et al., 2018) as did melittin against a polymicrobial biofilm consisting of selected dairy isolates of bacteria (*P. aeruginosa, Staphylococcus haemolyticus, K. pneumoniae*, and *Aeromonas caviae*) (Galdiero et al., 2019). The *de novo* AMP ASP-1 was able to eradicate polymicrobial biofilms containing *S, aureus, P. aeruginosa, A. baumannii*, and *K. pneumoniae* when formulated into a polyurethane-based dressing (Bayramov et al., 2018) and the Komodo dragon-derived peptide DRGN-1 demonstrated moderate eradication activity against mixed biofilms containing *P. aeruginosa* and *S. aureus* (Chung et al., 2017). Luo and co-workers tested a library of peptoids for antibiofilm activity and demonstrated that 3 peptoids from the library were able to eradicate mixed species biofilms (formed for 8 h) containing *C. albicans* and *S. aureus* or *C. albicans* and *E. coli* (Luo et al., 2017).

In general, concentrations of AMP required to permeabilise membranes in biofilms are higher than for their planktonic counterparts and this may reflect sequestration (for example, by extracellular DNA or other extracellular polymeric substances), hydrolysis of AMP in the biofilm, increased microbial cell density or may reflect changes to individual microorganisms residing within the biofilm, as bacteria growing in biofilms can have altered membrane permeability (Orazi and O'Toole, 2019), which may affect the activity of membrane-active AMP. Membranes of *S. aureus, Listeria monocytogenes, P. aeruginosa, Salmonella* Typhimurium growing as biofilms contain significantly higher proportions of saturated fatty acids compared to cells growing planktonically. Increases in saturated fatty acids (in particular long-chain fatty acids are concomitant to decreases in branched-chain fatty acid content, which can lead to increased membrane rigidity and stability (Denich et al., 2003; Dubois-Brissonnet et al., 2016).

Approved standards exist for AST of both bacteria and fungi (Table 1), but these apply only to microbes growing planktonically. Approved AST standards for microorganisms growing as biofilms do not exist and a number of different methods are used (Macia et al., 2014; Magana et al., 2018), making direct comparisons of different studies difficult to undertake. As described above, microorganisms growing as biofilms are more tolerant of antimicrobials than microbes growing planktonically, so the results of conventional AST cannot be used to accurately predict the results of biofilm AST (Macia et al., 2014). As an added level of complexity, the level of biofilm antimicrobial tolerance may also be influenced by the methods used to establish and monitor the biofilms.

At present it is not possible to specify an optimal testing procedure for assessing the activity of AMP against biofilms, given their heterogenous nature and the complexity underlying their development (Haney et al., 2018a; Magana et al., 2018). When reporting activity of AMP vs. biofilms it is vital, initially, to specify whether biofilm prevention or eradication is being described. When describing the anti-biofilm properties of AMP appropriate methodological details must be provided, including device used to grow the biofilm and the parameters used to establish anti-biofilm activity. Most commonly, biofilms are grown in multi-well plates or the Calgary device, which are simpler and cheaper than flow-cell devices and better suited to routine AST of biofilms (Macia et al., 2014). The most commonly used are the minimal biofilm inhibitory concentration (MBIC), defined as the lowest concentration of an antimicrobial at which there is no time-dependent increase in the mean number of biofilm viable cells (Moskowitz et al., 2004), or the minimal biofilm-eradication concentration (MBEC), defined as the lowest concentration of antibiotic required to eradicate the biofilm (Ceri et al., 1999). In the case of membrane-active AMP, evidence of membrane permeabilization must be provided, for example, the use of fluorescence microscopy (standard, confocal laser scanning microscopy or scanning electron microscopy) using membrane-impermeant fluorophores that allow demonstration of membrane permeabilization, including PI (Boulos et al., 1999) or Sytox dyes (Roth et al., 1997) or reporter genes. Recently, Haney and co-workers proposed two quick, easy, reproducible and inexpensive methods; one for assessing biofilm inhibition and the other for assessing biofilm eradication, to determine the activity of AMP against biofilms (Haney et al., 2018b). Both methods were based on the use of microtitre plates for detection and utilised the crystal violet protocol for determining biofilm biomass (O'Toole and Kolter, 1998; O'Toole, 2011) and in the eradication assay they assessed metabolic activity within the biofilm using the tetrazolium chloride dye (triphenyl tetrazolium chloride) (Brown et al., 2013; Sabaeifard et al., 2014). These methods are similar to many reported in the literature and appear suitable for simplified analysis of biofilm prevention and eradication and should also be readily adapted for use in the analysis of fungal biofilms, but do not provide an assessment of the effects of AMP on the bacterial membrane, which would require use of some of the methods outlined above.

The efficacy of AMP vs. bacterial and fungal spores has been the subject of a relatively limited number of studies. Bacterial endospores with relevance to human infectious diseases are mainly produced by *Clostridiodes* spp. (*Clostridium* spp.) and *Bacillus* spp. Bacterial endospores are metabolically dormant and environmentally resistant and are capable of surviving antibiotic exposure, extremes of temperature, desiccation and ionizing radiation (Higgins and Dworkin, 2012). The cathelicidin family AMP PG-1, BMAP28, and LL-37 demonstrated sporicidal activity against *B. anthracis* spores in the low mg/L range, whereas SMAP-29, CAP-18 were not effective and NA-CATH and mCRAMP demonstrated activity only at high concentrations (1,000 mg/L) (Blower et al., 2018). The AMP chrysophin-3 also demonstrated sporicidal activity vs. *B. anthracis* (Pinzon-Arango et al., 2013) and activity of PG-1 vs. spores of *B. anthracis* had been previously reported (Lisanby et al., 2008). The AMP TC19, TC84, BP2 and the lantibiotic nisin A were tested for efficacy against spores of *Bacillus subtilis* and were bactericidal against germinated spores by perturbing the inner membrane, thus preventing outgrowth to vegetative cells, but were not able to prevent germination (Omardien et al., 2018). Presumably, this is because the inner membrane of endospores is immobile and becomes fluid only during spore germination (Setlow, 2006). Other than this, the only peptides with activity vs. spores are the lantibiotics nisin (Gut et al., 2008) and subtilin (Liu and Hansen, 1993), both of which were only active against germinated spores, as above. Assessing the efficacy of AMP vs. bacterial spores for AST is relatively simple, by substituting a spore suspension for the normal inoculum, but in order to fully understand the precise effect of AMP on spores requires more specialised techniques, such as those described by Omardien et al. (2018). As spores are not normally the cause of the disease, rather this is vegetatively growing cells, the importance of AMP activity against spores is of more relevance to disease prevention, rather than cure.

Activity specifically against fungal spores (conidia) has received limited attention compared to activity against vegetatively growing fungi or yeast. Fungal spores are often the infectious propagule that initiates disease, e.g., inhalation of *Aspergillus* spp. spores into the lung, but the disease is caused once the spores germinate and the fungi begin vegetative growth (Kosmidis and Denning, 2015). As with efficacy vs. bacterial endospores, sporicidal activity is of more relevance to disease prevention than cure. Nevertheless, activity against vegetatively growing cells and spores is a desirable property for any antifungal AMP. The thaumatin-like protein osmotin demonstrated antifungal activity against fungal hyphae of predominantly plant-pathogenic fungi and caused lysis of spores of *Fusarium moniliforme, F. oxysporum, Trichoderma longibrachatum*, and *Verticillium dahlaie* (Abad et al., 1996) as did the synthetic undecapeptides Pep3, BP15, BP20, BP33, and BP76 vs. *F. oxysporum* conidia (Badosa et al., 2009). The antifungal peptide drosomycin, originally isolated from *Drosophila* spp., inhibited fungal spore germination (Zhang and Zhu, 2009) as did bombinins H2 and H4 which were active against spores of Phytophthora nicotianae (Matejuk et al., 2010). Peptide KK14 and derivatives inhibited spore germination of *F. culmorum, Penicillium expansum*, and *A. niger*, albeit with reduced activity vs. germinated conidia (Thery et al., 2019) as did the AMP O_3_TR and derivatives (Thery et al., 2018). Rabbit neutrophil cationic peptides, however, were not active against ungerminated spores of *Aspergillus fumigatus* or *Rhizopus oryzae* (Levitz et al., 1986). When carrying out broth dilution AST using filamentous fungi, the inoculum used is a spore (conidial) suspension, but no attempt is made to determine whether the antifungal used prevents spore germination or is solely active against germinated spores/hyphae. The measure of inhibition is optical density or a visual (i.e., not using microscopy) determination of growth/no growth (CLSI, 2017c; EUCAST, 2017b), except in the case of echinocandins using EUCAST methodology in which the Minimum Effective Concentration (MEC) endpoint is determined (EUCAST, 2017b). Thus, it is clear that echinocandins do not prevent spore germination (i.e., not sporicidal), but this cannot be determined for other antifungals, including AMP, without additional analyses, such as microscopic analysis over time (Abad et al., 1996). Alternatively, spores could be exposed to AMP for only limited time periods in which spore germination could not have taken place (i.e., killing activity can have affected only spores) followed by plating on non-selective media to determination sporicidal activity (Badosa et al., 2009).

Small colony variants (SCV) constitute a slow-growing sub-population of bacteria that are characterized by impaired growth, atypical colony morphology, and an ability to persist in mammalian cells. SCV are characterized by down-regulation of genes for metabolism and virulence, while genes important for adhesion, persistence and biofilm formation are often up-regulated (Proctor et al., 2006; Johns et al., 2015; Kahl et al., 2016). SCV are frequently deficient in electron transport (menadione and haemin auxotrophs) or thiamine biosynthesis (thymidine auxotrophs) and these phenotypes can be reversed by supplementation with menadione, haemin or thymidine, respectively (Proctor et al., 2006). Additionally, SCV are less susceptible to various antibiotics including aminoglycosides, such as tobramycin, trimethoprim-sulfamethoxazole, fluorquinolones, fusidic acid, and even to antiseptics like triclosan (Kahl, 2014; Evans, 2015; Bui et al., 2017). SCV are relatively commonly isolated from infections and are also responsible for latent or recurrent infections, often once antibiotic selection is removed, and are often present in chronic infections (Proctor et al., 2006; Johns et al., 2015). In a number of cases, intracellular SCV have been isolated from sites of infection, including fibroblasts, osteoblasts, macrophages and endothelial cells (Kahl et al., 2016) and intracellular presence can induce SCV formation (Vesga et al., 1996). SCV normally constitute a minor sub-population of cells at the site of infection and therefore their isolation in sufficient numbers for the purposes of AST can be challenging (Johns et al., 2015; Kahl et al., 2016). SCV have been identified in diverse range of bacterial genera and isolated from clinical specimens, including *S. aureus, S. epidermidis, S. capitis, P. aeruginosa, Salmonella* serovars, *Burkholderia* spp., *Vibrio cholerae, Shigella* spp., *Brucella melitensis, E. coli, Lactobacillus acidophilus, Serratia marcescens*, and *Neisseria gonorrhoeae* (Proctor et al., 2006; Johns et al., 2015; Kahl et al., 2016). At present, there are no approved methods for AST of SCV, and *S. aureus* SCV do not grow well in CA-MH broth, making it difficult to generate meaningful results from broth-dilution AST (Precit et al., 2016). Precit and co-workers developed a disk diffusion method suitable for AST of *S. aureus* SCV (Precit et al., 2016), but this method is unlikely to work well with AMP due to the know interactions of cationic AMP with the negatively charged sulphate and sugar components of the agaropectin in agar (Lehrer et al., 1991). Another difficulty when working with SCV is their reversion to a normal colony phenotype (Proctor et al., 2006; Johns et al., 2015; Kahl et al., 2016). Other studies have described AST of SCV (see Table S1 in Precit et al., 2016 for a complete list), including direct use of the CLSI broth dilution procedure (Gao et al., 2010; Singh et al., 2010) and modifications of this, including prolonged incubation times (von Eiff et al., 1997; Samuelsen et al., 2005; Mercer et al., 2017) and media changes (e.g., Brain Heart Infusion (BHI) Kahl et al., 1998; Yagci et al., 2013). Naturally, AST of intracellular SCV adds a further level of complexity and the authors are not aware of any studies on the activity of AMP against intracellular SCV. For experimental purposes, SCV of *S. aureus* (with the characteristics of clinical SCV) can be selected for *in vitro* by incubation in the presence of the *P. aeruginosa* quorum sensing molecule 4-hydroxy-2-heptylquinoline-*N*-oxide (HQNO) (Hoffman et al., 2006), although the authors are not aware whether this works with other staphylococci or other bacterial genera. It was reported that SCV of *E. coli* could be generated following exposure to 2-methyl-1,4-napthoquinone (Colwell, 1946) or copper ions (Weed and Longfellow, 1954) and that mutants in a number of bacterial genes can generate SCV (Santos and Hirshfield, 2016; Tikhomirova et al., 2018; Al Ahmar et al., 2019; Vidovic et al., 2019). The importance of SCV in the context of infection means that AMP activity vs. SCV could represent an important method for the treatment of chronic, recurrent and antibiotic resistant infections, but has received only limited attention in the context of AMP. Glaser and co-workers demonstrated that SCV of *S. aureus* were less susceptible to the skin-derived AMP RNase7, hBD-2, hBD-3, and LL-37 than wild-type *S. aureus* as was a *hemB* mutant when compared to the complemented mutant, with the exception of LL-37. SCV were also less susceptible to the killing activity of human stratum corneum (Glaser et al., 2014). Similarly, SCV of *P. aeruginosa* were also less susceptible to LL-37 than the wild-type (Pestrak et al., 2018). *S. aureus* SCV were less susceptible to the AMP lactoferricin B and thrombin-induced microbicidal protein (tPMP) when compared to the wild-type and in the case of lactoferricin B, this was irrespective of the underlying auxotrophy (Koo et al., 1996; Samuelsen et al., 2005). Conversely, an antimicrobial polypeptide (NP108) retained comparable efficacy vs. *S. aureus* SCV when compared to wild-type *S. aureus* (Mercer et al., 2017). In another study, *S. aureus* SCV were less susceptible to the AMP protamine, but equally susceptible to magainin and HNP-1, whereas SCV were more susceptible to dermaseptin than the wild-type *S. aureus*. Interestingly, exposure to sub-MIC concentrations of protamine selected for SCV *in vitro* (Sadowska et al., 2002). Zhang and co-workers reported that wild-type and SCV of *S. aureus* were equally susceptible to LL-37, but that SCV became less susceptible in the presence of bicarbonate, adding a further level of complexity of AST of SCV (Zhang et al., 2018). The above results appear to indicate that susceptibility of SCV of *S. aureus* to AMP needs to be determined on a case-by-case basis and potentially that he method of AST adopted is also important.

Persister cells are dormant or slow-growing variants of normal wild-type cells, forming a sub-population that are highly tolerant to antimicrobials (despite no genetic basis for resistance), but that can revert back to wild-type growth and sensitivity. Persister cells can be responsible for recalcitrance of infections and relapse following treatment and the emergence of antibiotic resistance (Fisher et al., 2017; Balaban et al., 2019). Most work to-date has been carried out on bacterial persister cells, however, fungal persister cells have been isolated from biofilms and that share many of the characteristics of bacterial persisters, including tolerance to high doses of antifungals (Bojsen et al., 2017; Wuyts et al., 2018). Given the importance of persister cells in infection, it is important to develop antimicrobial agents capable of eradicating persister cells and the efficacy of AMP against bacterial persister cells has been examined in a limited number of studies. The dendrimeric AMP, 2D-24, was active against persister cells of *P. aeruginosa* PAO1 and a mucoid mutant PDO300, as well as planktonic and biofilm cells (Bahar et al., 2015). Trp/Arg-containing AMP successfully killed planktonic and biofilm persister cells of *E. coli* (Chen et al., 2011), and the aryl-alkyl-lysine NCK-10 against persister cells, planktonic cells and biofilms of *S. aureus* (Ghosh et al., 2015) and the LL-37 derivative SAAP-148 (de Breij et al., 2018). Additionally, the temporin analogue, TB_L1FK, the β-defensin derivative, C5, and the dendrimeric AMP, Den-SB056, were equally effective against *in vitro* generated persister and wild-type cells of *P. aeruginosa* and *S. aureus* (Grassi et al., 2017b). Persister cells of some bacterial species can be generated in the laboratory by antibiotic exposure (Dorr et al., 2009; Sulaiman et al., 2018) or treatment with specific chemicals, including *E. coli, P. aeruginosa*, and *S. aureus* using the uncoupling agent carbonyl cyanide m-chlorophenyl hydrazone (CCCP) (Kwan et al., 2013; Grassi et al., 2017a) and *E. coli* using salicylate (Wang et al., 2017), facilitating AST of persisters with AMP.

### Interactions of AMP With Antibiotics/Antifungals

A large number of publications have described *in vitro* synergy of selected AMP with other AMP (Zerweck et al., 2017; Hanson et al., 2019), antibiotics (for example Cassone and Otvos, 2010; Sakoulas et al., 2014; Soren et al., 2015; Pollini et al., 2017; Pizzolato-Cezar et al., 2019) or antifungals (Duggineni et al., 2007; Singh et al., 2017), although this is not always the case (He et al., 2015) and is something that is no doubt under-reported. Conjugates of AMP and antibiotics, organometallic compounds, gold nanoparticles or to create AMP polymers to increase efficacy, to reduce toxicity and/or to improve formulation have become of increasing interest over recent years (for examples, see Reinhardt and Neundorf, 2016; Rajchakit and Sarojini, 2017; David et al., 2018; Sun et al., 2018) and will undoubtedly require additional consideration when it comes to AST. *In vitro* synergy is often species- or even strain-specific and is often specific to certain antimicrobials, so there are no general rules with respect to predicting synergy. Whilst *in vitro* synergy between antibiotics or antifungals demonstrates potential, there is limited evidence that synergy translates into the clinic, beyond β-lactam/β-lactamase combinations such as piperacillin/tazobactam and ceftazidime/avibactam, so perhaps it might be set to temper this enthusiasm until detailed standardisation of testing methods and clinical trials have been carried out (Doern, 2014). When conducting AST with combinations of AMP with conventional antimicrobials care needs to be taken analysing antimicrobials where solvent other than water are used (e.g., DMSO), or where conventional media requires supplements, including blood, NaCl and polysorbate (see above for the potential effects of these compounds on AMP), as they may impact upon AMP activity. Dimethyl sulfoxide (DMSO) can function as a membrane permeabiliser and can depolarize membranes (Yu and Quinn, 1998) and clearly, this may enhance the apparent activity of membrane permeabilising AMP.

## AMP in Clinical Development

A limited number of antibiotics comprising a peptide element are in clinical use (Table 4), but as yet, no AMP have been approved as therapies although a number are in clinical trials (Table 5). Koo and Seo, in a comprehensive review of AMP drug development, state that approximately half of AMP currently in development are at the preclinical stage, while a third have moved forward to clinical trials, most of those are currently in phase II. Approximately 15% of the AMP failed during one of these stages (Koo and Seo, 2019). These numbers are much more promising than the ones presented by Lau and Dunn for previous years, where the number of discontinued AMP trials was higher than 50% (Lau and Dunn, 2018). To increase the clinical pipeline of AMP drug candidates and more importantly, approval of these potentially AMR game-changing antimicrobials, better and more appropriate AST (and *in vivo* models, although this not a topic for this manuscript) is critical.

**Table 4:** Clinically approved peptide antimicrobials.

**Antimicrobial**	**Drug name**	**Company**	**Class**	**Source**	**Mol Wt**	**Application**	**MOA**	**Dosing**	**References**
Colistin (Polymyxin E)	Coly-Mycin	Generic	Lipopeptide	*Bacillus colistinus*	1155.4	Antibacterial; Gram -	Membrane disruption	IV, IM and Inhalation	Karaiskos et al., 2017
Dalbavancin^a^	Dalvance	Durata Therapeutics	Lipoglycopeptide	Semi-synthetic	1816.7	Antibacterial; Gram +	Cell wall biosynthesis	IV	Bassetti et al., 2018
Daptomycin	Cubicin	Merck (Cubist)	Lipopeptide	*Streptomyces roseosporus*	1620.6	Antibacterial; Gram +	Membrane disruption	IV	Gonzalez-Ruiz et al., 2016
Gramicidin D	NA	Generic	Linear peptides	*Bacillus brevis*	1882.2	Antibacterial; Gram +	Membrane disruption	Topical	Burkhart et al., 1999
Gramicidin S	NA	Generic	Cyclic peptide	*Bacillus brevis*	1141.4	Antibacterial; Gram + and -	Membrane disruption	Topical	Mogi and Kita, 2009
Oritavancin^b^	Orbactiv	Melinta Therapeutics	Lipoglycopeptide	Semi-synthetic	1793.1	Antibacterial; Gram +	Cell wall biosynthesis	IV	Saravolatz and Stein, 2015
Polymyxin B	NA	Generic	Lipopeptide	*Bacillus polymyxa*	1203.5	Antibacterial; Gram -	Membrane disruption	IV and IM	Rigatto et al., 2019
Teicoplanin	Targocid	NPS Pharma	Glycopeptide	*Actinoplanes teichomyceticus*	1879.6	Antibacterial; Gram +	Cell wall biosynthesis	IV and IM	Campoli-Richards et al., 1990
Telavancin^b^	Vibativ	Theravance	Lipoglycopeptide	Semi-synthetic	1755.7	Antibacterial; Gram +	Cell wall biosynthesis	IV	Higgins et al., 2005
Vancomycin	NA	Generic	Glycopeptide	*Streptomyces orientalis*	1449.3	Antibacterial; Gram +	Cell wall biosynthesis	Oral and IV	Alvarez et al., 2016
Anidulafungin	Eraxis	Pfizer	Echinocandin (lipopeptide)	Semi-synthetic	1140.2	Antifungal	Cell wall biosynthesis	IV	Mayr et al., 2011
Caspofungin	Cancidas	Merck	Echinocandin (lipopeptide)	Semi-synthetic	1093.3	Antifungal	Cell wall biosynthesis	IV	Song and Stevens, 2016
Micafungin	Mycamine	Astellas	Echinocandin (lipopeptide)	Semi-synthetic	1270.3	Antifungal	Cell wall biosynthesis	IV	Scott, 2012

IV, Intravenous; IM, Intramuscular; NA, Not applicable;

a Dalbavancin is a semi-synthetic derivative of Teicoplanin;

b *Oritavancin and Telavancin are semi-synthetic derivatives of Vancomycin*.

**Table 5:** AMP in clinical development (adapted from Koo and Seo, 2019).

**AMP**	**Source**	**Target^c^**	**Phase**	**Company**	**Administration^d^**
EA-230	hCG derivative	Sepsis and renal failure protection	II	Exponential biotherapies	Iv
CZEN-002	α-MSH derivative	Anti-fungal	II^b^	Zengen	Top
D2A21	Synthetic	Burn wound infections	III	Demegen	Top
XMP-629	BPI derivative	Impetigo and acne rosacea	III^b^	Xoma Ltd.	Top
Neuprex(rBPI21)	BPR derivative	Peadiatric meningococcemia	III^b^	Xoma Ltd.	iv
Delmitide(RDP58)	HLA class I derivative	Inflammatory bowel disease	II^a^	Genzyme	Top
Ghrelin	Endogenous HDP	Chronic respiratory failure	II^a^	University of Miyazaki; Papworth Hospital	iv
hLF1-11	Lactoferricin derivative	MRSA, *K. pneumoniae, L. monocytogenes*	I/II	AM-Pharma	iv
C16G2	Synthetic	Tooth decay by *Streptococcus mutans*	II	C3 Jian Inc.	Mouthwash
SGX942(Dusquetide)	Synthetic	Oral mucositis	III	Soligenix	Oral rinse
DPK-060	Kininogen derivative	Acute external otitis	II	ProMore pharma	Ear drops
PXL01	Lactoferrin analogue	Postsurgical adhesions	III	ProMore pharma	Top
PAC113	Histatin 5 analogue	Oral candidiasis	II^a^	Pacgen biopharmaceuticals	Mouth rinse
POL7080	Protegrin analogue	*P. aeruginosa, K. pneumoniae*	III	Polyphor Ltd.	iv
LTX-109 (Lytixar)	Synthetic	G+, MRSA skin infection; impetigo	II^a^	Lytix biopharma	Top
OP-145	LL-37 derivative	Chronic middle ear infection	II^a^	Dr. Reddy's research	Ear drops
LL-37	Human cathelicidin	Leg ulcer	II^b^	ProMore pharma	Top
Novexatin (NP213)	Cyclic cationic peptide	Fungal nail infection	II^a^	NovaBiotics Ltd.	Top
p2TA (AB103)	Synthetic	Necrotizing soft tissue infections	III	Atox Bio Ltd.	iv
Iseganan (IB-367)	Protegrin analogue	Pneumonia, stomatitis	III^b^	IntraBiotics pharmaceuticals	Top
Pexiganan (MSI-78)	Magainin analogue	Diabetic foot ulcers	III^b^	Dipexium pharmaceuticals	Top
Omiganan (CLS001)	Indolicidin derivative	Rosacea	III	Cutanea life sciences	Top

a *Clinical trial completed*.

b *Clinical trial discontinued*.

c *Target microorgansim: G+ - Gram positive; MRSA – methicillin-resistant S. aureus*.

d *Route of administration: top - topical; iv – intravenous*.

## Conclusions

Antimicrobial resistance is a global health problem that will require the survey of a large number of molecules with a variety of chemistries and new functional families. AMP are clearly a group of molecules with significant potential as a new class of therapy to address the urgent need for better and AMR-mitigating antibacterial and antifungal therapies. Among other advantages highlighted in this and other reviews, AMP offer great versatility in terms of chemical functionality. Unfortunately, “standard” AST protocols can significantly underestimate the efficacy of AMP and improved, more predictive methods are required to identify leads and facilitate faster progress of AMP into pre-/clinical development. These changes to “standard” protocols will require rigorous and detailed justification and a degree of validation. New AST of AMP will have to take into account that many AMP depend on their positive charge for activity as well as the many other factors we have enumerated in this review (see Table 2). Despite the manifold factors that influence AMP activity, we are confident that research toward developing more appropriate AST for AMP is headed in the right direction. New protocols will, in the near future, accelerate the discovery process of novel AMP therapeutics.

## Author Contributions

DM, SD, MT, EL, LS, MK-B, CF-N, DO'N, and AA-B contributed to the writing of this manuscript. DM, MT, CF-N, DO'N, and AA-B contributed to the editing of this manuscript. All authors contributed to the article and approved the submitted version.

## Conflict of Interest

DM, EL, LS and DO'N are employed by NovaBiotics Ltd. The remaining authors declare that the research was conducted in the absence of any commercial or financial relationships that could be construed as a potential conflict of interest.
